# Integrating Transformer Segmentation and Morphological Analysis for Multi-Crack Characterization and Visual Equivalent Strain Assessment in Rubberized ECC

**DOI:** 10.3390/polym18212657

**Published:** 2026-10-26

**Authors:** Junqing Jia, Tengxiang Su, Jinghao Huang, Ge Zhang, Jiaxiang Lin

**Affiliations:** 1 Guizhou Power Grid Co., Ltd., Guiyang 550029, China; 2 School of Civil and Transportation Engineering, Guangdong University of Technology, Guangzhou 510006, China

**Keywords:** engineered cementitious composites, crack segmentation, lightweight transformer, crack morphology, visual equivalent strain, large language model

## Abstract

Deep-learning semantic segmentation and morphological analysis offer a non-contact route to characterizing microcracks in cementitious composites, yet linking pixel-level masks to physical geometry and tensile response remains challenging. This study establishes an integrated lightweight segmentation and geometric-quantification workflow for rubberized engineered cementitious composites (ECCs) incorporating 0–50% powdered rubber. We propose EfficientSegLite, a supervised binary segmentation architecture combining a hierarchical SegFormer-B0 encoder, a channel-compressed U-Net-style decoder, lightweight atrous spatial pyramid pooling, and depthwise separable convolutions, trained via a two-stage Dice–OHEM–Edge strategy. On 1440 ECC tensile images, it achieved 93.40% mIoU and 93.24% F1 score with only 3.96 M parameters and 734.9 M FLOPs. Deterministic morphological and distance-transform algorithms then convert the masks into calibrated physical descriptors, achieving a mean relative crack-width error of 3.65% against optical microscopy across eight benchmark cracks. A crack-area-based visual equivalent strain (ε_v) correlated strongly with extensometer strain (Pearson’s *r* > 0.97, *R*^2^ > 0.94); however, regression slopes of 0.368–1.046 and uncalibrated mean absolute errors of 0.03–2.04 percentage points indicate that quantitative strain estimation requires mixture-specific calibration. A tool-calling large language model agent parses user instructions and orchestrates the deterministic computation tools, generating traceable inspection reports.

## 1. Introduction

Engineered cementitious composite (ECCs) is a high-performance fiber-reinforced cementitious material [1] characterized by pseudo strain hardening and multiple cracking [2,3]. Its tensile strain capacity can reach 3–8%, accompanied by numerous microcracks during loading [4]. Crack widths generally remain below 100 μm [5,6,7]. These distributed microcracks provide ECC with high ductility and crack resistance and can extend structural service life [8,9,10,11,12]. However, crack evolution still affects the long-term durability of ECC, particularly its resistance to corrosion and water penetration [13,14]. Accurate detection and quantification of ECC microcracks are therefore essential for material-performance evaluation and structural-safety assessment.

Recent material studies show that mixture design and exposure conditions alter ECC responses in ways that matter for crack characterization. Water-to-binder and sand-to-binder ratios affect the dynamic compressive response of seawater coral sand ECC [15], while water-to-binder ratio and particle size affect the dynamic behavior of ECC with recycled polyethylene terephthalate (PET) fine aggregate [16]. Recycled PET aggregates can also promote crack activation and tensile ductility by acting as engineered flaws [17], while the crack-width distribution of PET-modified high-strength, high-ductility composites changes under sub-elevated temperatures [18]. These findings motivate quantitative crack assessment across ECC compositions and conditions.

Conventional crack-detection techniques include nondestructive methods such as ultrasonics [19], infrared thermography [20], and electrical sensing [21,22]. These methods often require substantial manual intervention and are sensitive to environmental conditions and subjective judgment. Image-processing approaches based on edge detection [23], threshold segmentation [24], and denoising [25] are sensitive to image quality. They can produce biased results under blur, noise, or nonuniform illumination and may fail to quantify microcracks accurately. Deep learning has shifted crack detection from handcrafted features toward data-driven methods [26,27,28,29,30]. Semantic segmentation networks such as convolutional neural networks (CNNs) and U-Net have been widely applied to crack detection [31,32,33]. Cha et al. [34] used a CNN to segment pavement cracks and outperformed conventional edge detection in complex backgrounds. U-Net and Mask R-CNN can segment macrocracks but do not adequately model the long-range dependencies of slender ECC cracks. The resulting predictions may therefore be discontinuous. transformer models establish global relationships through self-attention and improve segmentation in complex backgrounds [35]. Wang et al. [36] proposed SegCrack, which uses a hierarchical transformer to capture long-range crack dependencies. Guo et al. [37] developed a transformer-based network with an attention mechanism for automatic pavement crack detection. Nevertheless, high-accuracy transformer models often require many parameters and substantial computation, limiting their use for ECC microcrack detection.

Crack segmentation connects visual perception with material-mechanics analysis. Previous studies have extracted crack length, width, area, and topological features from segmented masks using skeletonization, distance transforms, and morphological operations. Kang et al. [38] combined region detection, pixel-level segmentation, and an improved distance transform to measure the length and thickness of concrete cracks in complex backgrounds. Carrasco et al. [39] measured surface-crack width from a distance transform and crack skeleton and characterized width variation along the crack path. Li et al. [40] combined contour extraction, skeletonization, and scale calibration to calculate the length, width, and area of asphalt-pavement cracks. Chen et al. [41] integrated pixel-level segmentation with three-dimensional reconstruction to locate and quantify complex concrete cracks in semantic point clouds. These methods mainly address cracks on ordinary concrete, pavements, or other structural surfaces and often quantify only length and width. Their results are also sensitive to segmentation error, image resolution, and scale calibration. A unified framework that combines lightweight segmentation, physical quantification, and mechanical characterization remains unavailable for the narrow, numerous, and densely distributed microcracks in ECC. A complete workflow is therefore needed to connect image-based detection with geometric measurement and engineering interpretation. Table 1 compares representative studies and the scope of the present workflow.

The studies use different materials, datasets, imaging conditions, and evaluation protocols. This comparison summarizes methods and scope; it is not a ranking of accuracy under a common test protocol.

Large language models (LLMs), including OpenAI GPT, Google Gemini, and Anthropic Claude, provide natural-language interfaces for intelligent analysis. General-purpose LLMs, however, cannot directly perform reliable quantitative calculations of crack width and length. Vision-language models have been applied to construction-image captioning, daily report generation [44] and zero-shot monitoring of personal protective equipment [45]. Other applications include ontology-enhanced agents for construction safety [46] and safety-state classification and explanation [47]. Multimodal models have also been explored for structural-damage identification and natural-language description [48]. These studies primarily address qualitative tasks such as classification, image description, or visual question answering. Traceable crack quantification requires LLMs to be coupled with deterministic segmentation and geometric-analysis tools.

Three limitations remain in current approaches. First, high-performance segmentation models are computationally expensive, making it difficult to balance microcrack accuracy with deployment efficiency. Second, crack pixels occupy only a small fraction of ECC images, and generic loss functions often fail to delineate fine boundaries accurately, introducing errors in width measurements. Third, most methods stop at crack detection or segmentation and do not provide a closed workflow from semantic masks to geometric parameters and mechanical indicators.

To address these challenges, this study develops an integrated framework centered on EfficientSegLite, a lightweight supervised deep-learning architecture for pixel-wise binary crack segmentation, coupled with deterministic morphological quantification and mechanical correlation for rubberized ECC. While the foundational operators—such as hierarchical transformer self-attention, U-Net skip connections, atrous spatial pyramid pooling, depthwise separable convolutions, and focal hard-example mining—are established in computer vision, the technical contribution of this work lies in their task-specific architectural synergy for ultra-thin, sparse ECC microcracks and their closed-loop integration with physical and micromechanical characterization:

(1) Lightweight multiscale architecture for fine microcracks: To overcome the coarse boundary recovery of standalone SegFormer-B0 (which yields only 59.71% mIoU due to 4× bilinear upsampling) and the high computational overhead of standard U-Net/ASPP hybrids (4.659 G FLOPs), EfficientSegLite introduces 1 × 1 channel compression along shallow skip pathways, places a single Light-ASPP module exclusively at the deepest semantic bottleneck, and reconstructs multi-level features using OptimizedDWConv blocks. This design reduces computational complexity by 84.2% (to 734.9 M FLOPs) and parameters to 3.96 M while achieving 93.40% mIoU and 93.24% F1 score.
(2) Two-stage Dice–OHEM–Edge optimization: A two-stage curriculum dynamically shifts loss weights from global regional overlap (Dice + Focal loss in Stage 1) to hard boundary pixels and topological continuity (incorporating OHEM and a Sobel L1 edge penalty in Stage 2). Repeated training evaluations confirm that this strategy significantly reduces run-to-run variance in mIoU and precision, suppressing false positives caused by cementitious matrix textures and surface pores.
(3) End-to-end physical quantification and visual equivalent strain analysis: Deterministic image-processing routines map binary masks into calibrated physical crack area, skeleton length, distance-transform width, tortuosity, and fractal dimension across rubberized ECC mixtures (0–50% replacement). A crack-area-based visual equivalent strain (*ε_v_*) is evaluated through both linear regression and error/agreement metrics (RMSE, MAE, and Bland–Altman analysis), clarifying both its capability for non-contact damage tracking and the physical calibration required for macroscopic strain estimation.
(4) Traceable tool-calling LLM inspection system: A decoupled inspection system combines a PyQt5 local computation platform with a DeepSeek-based LLM agent. All numerical crack descriptors are computed strictly by deterministic algorithms, whereas the LLM agent handles natural-language instruction parsing, tool invocation, and structured engineering report generation.

## 2. Materials and Methods

**Figure 1:**
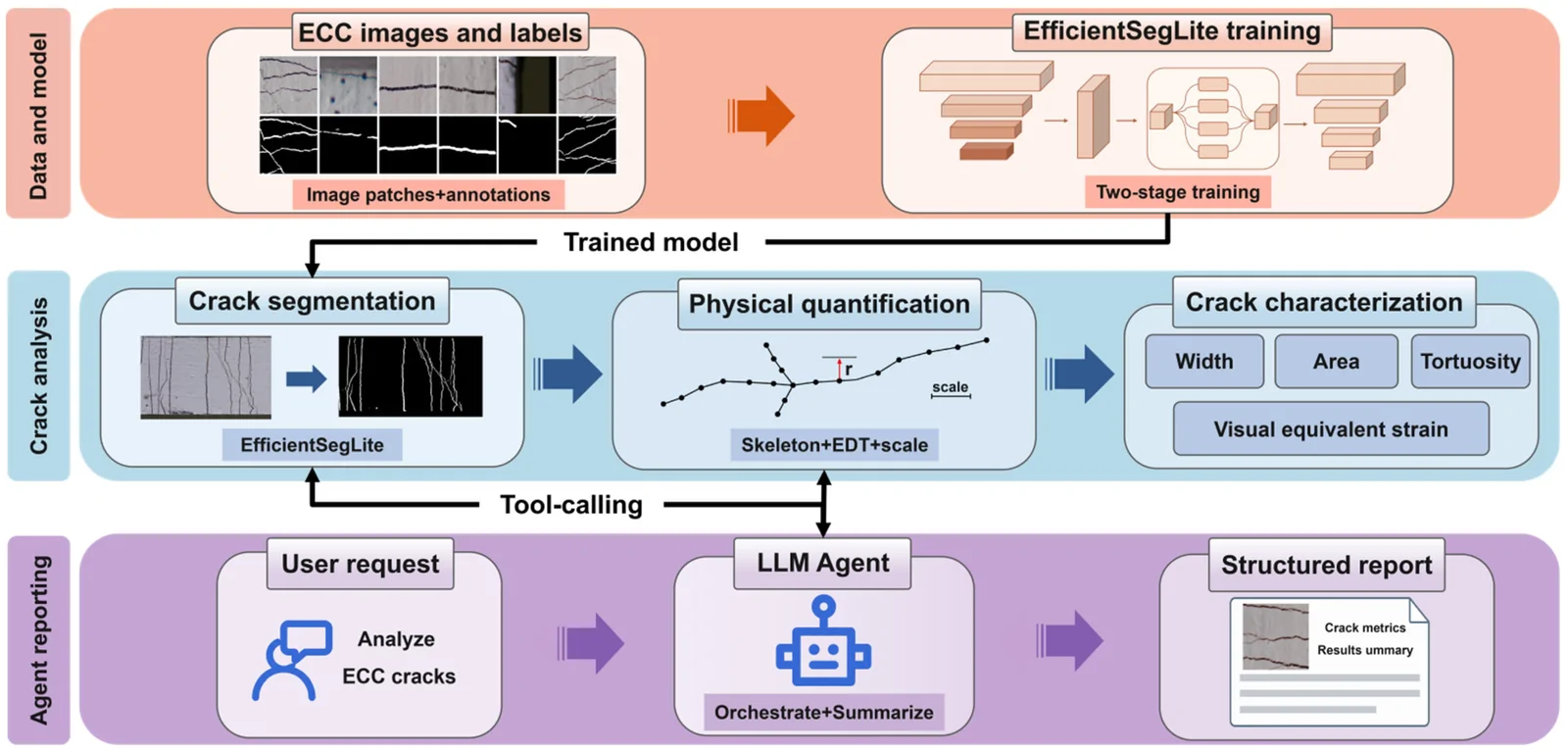
Overall workflow of the proposed method.

An image dataset was constructed from ECC uniaxial tensile tests, and a crack-segmentation model was designed using SegFormer-B0 and U-Net. Light-ASPP was introduced into the deepest feature stage, while DW-Conv reduced computation throughout the network. The model was then trained and optimized on the resulting dataset. Finally, the segmentation model was integrated with crack-quantification algorithms for automated parameter analysis. The overall workflow is shown in Figure 1.

### 2.1. Dataset Preparation

#### 2.1.1. Raw Materials

**Figure 2:**
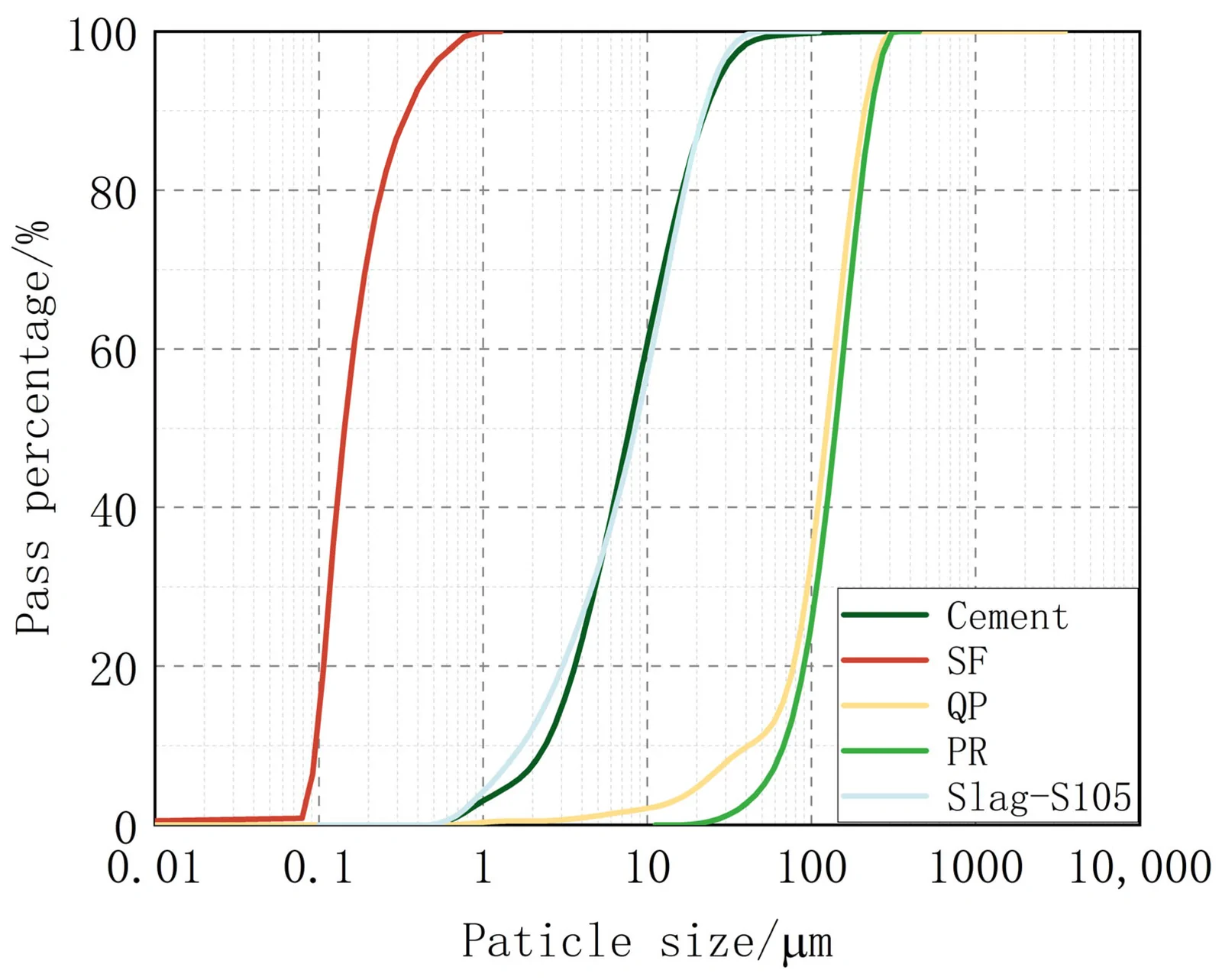
Particle-size distribution curves of the cement, GGBFS (Slag-S105), silica fume, quartz powder, and powdered rubber measured by laser diffraction.

The constituents of ECC are P.II 52.5R Portland cement, ground granulated blast-furnace slag (GGBFS), silica fume (SF), quartz powder (QP), powdered rubber (PR), ultra-high-molecular-weight polyethylene (PE) fibers, a polycarboxylate-based high-range water-reducing admixture (superplasticizer, SP), and water. Based on material characterization records, the particle-size ranges were 1–30 μm for cement, 1–25 μm for GGBFS, 0.1–1 μm for silica fume, and 50–300 μm for quartz powder. The specific surface areas were 429 m^2^/kg for GGBFS and 20,000–25,000 m^2^/kg for silica fume. Chemical composition analysis indicated that the silica fume contained ≥92% SiO_2_, while the oxide composition of GGBFS (by mass%) consisted of 34.50% SiO_2_, 34.00% CaO, 17.70% Al_2_O_3_, 6.01% MgO, 1.64% SO_3_, 1.03% Fe_2_O_3_, and 5.12% minor constituents. Waste tire powdered rubber with an apparent density of 1120 kg/m^3^ was used for partial volumetric replacement of fine aggregate, with a particle-size range comparable to that of the quartz powder (50–300 μm). The particle-size distribution curves of the cement, GGBFS (Slag-S105), silica fume, quartz powder, and powdered rubber measured by laser diffraction are shown in Figure 2. Full oxide percentages for the commercial cement batch were not separately archived. The PE fibers had a diameter of 20 μm, a length of 12 mm, a tensile strength of 3000 MPa, an elastic modulus of 116 GPa, and a density of 0.97 g/cm^3^ (Table 2). The polycarboxylate-based superplasticizer (solid content 40%) was applied at a constant dosage of 1.6% by mass of binder.

#### 2.1.2. Specimen Preparation

**Figure 3:**
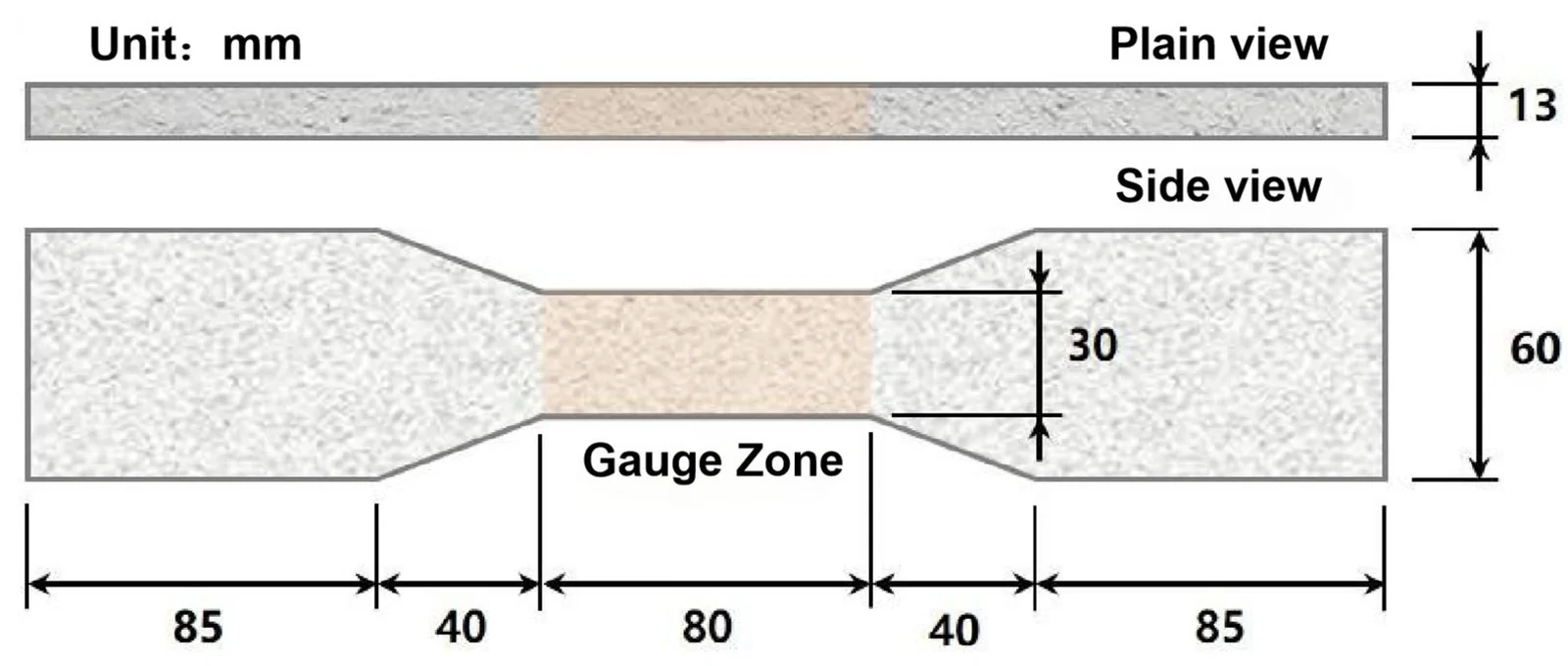
Geometry and dimensions of the dog-bone specimen.

Six rubberized ECC mixtures were designed by replacing quartz powder with powdered rubber at volumetric replacement ratios of 0%, 10%, 20%, 30%, 40%, and 50% (designated as 0–12 through 50–12, where 12 denotes the 12 mm PE fiber length), as detailed in Table 3. Following standard mixing and casting procedures, dog-bone tensile specimens measuring 330 mm × 60 mm × 13 mm with a central gauge zone of 80 mm × 30 mm × 13 mm (Figure 3) were fabricated following the Japan Society of Civil Engineers (JSCE) Recommendations for HPFRCC [49]. All specimens were demolded after 24 h and cured under standard laboratory conditions (20 ± 2 °C, ≥95% relative humidity) for 28 days prior to testing.

#### 2.1.3. Test Setup and Image Acquisition

**Figure 4:**
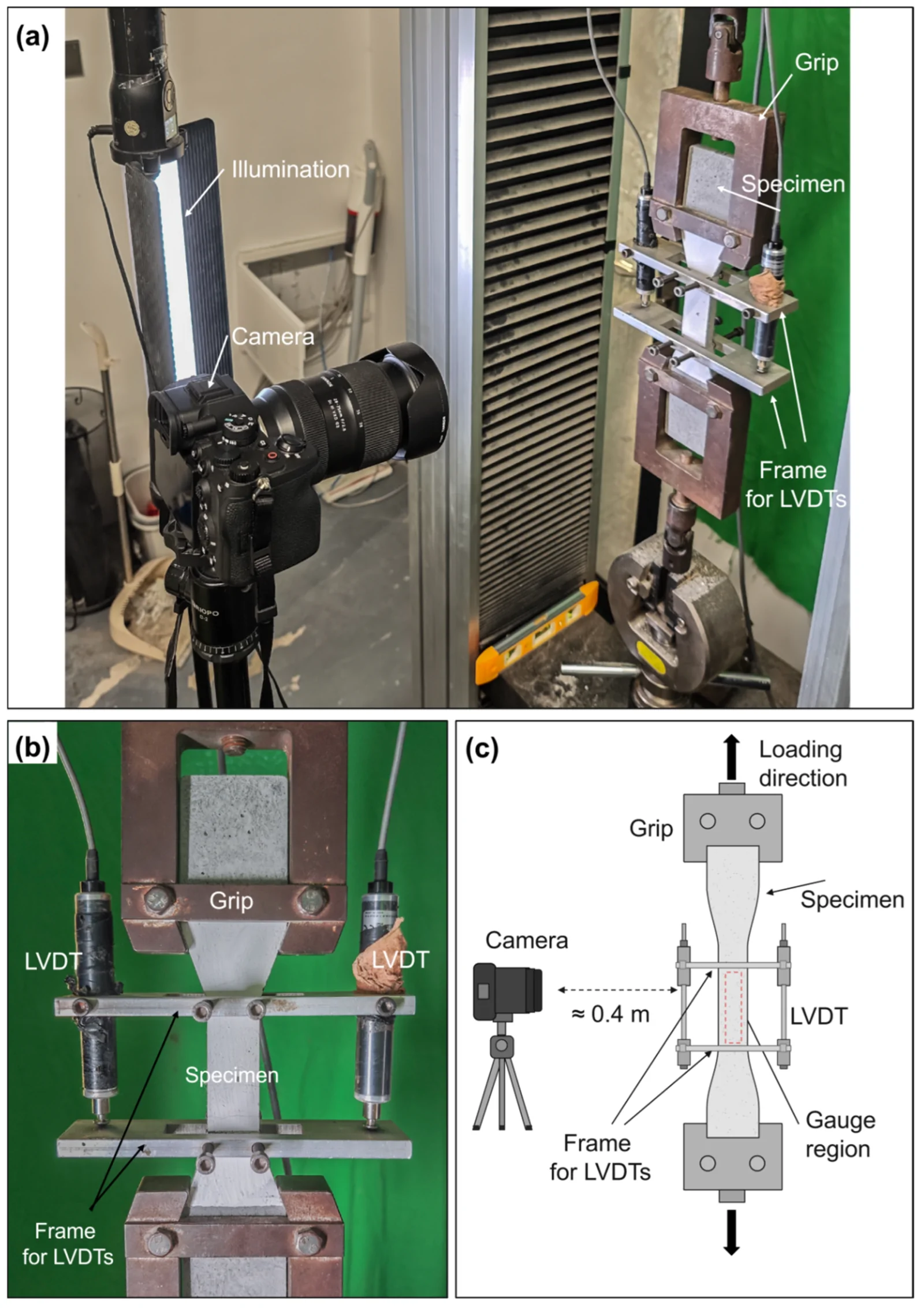
Experimental setup for the uniaxial tensile test and image acquisition of ECC specimens: (**a**) overall configuration of the testing machine, camera, and illumination system; (**b**) detailed view of the ECC dog-bone specimen and displacement measurement system; (**c**) schematic illustration of the image acquisition configuration and loading direction.

The ECC dog-bone specimens were subjected to uniaxial tension in a universal testing machine at 0.5 mm/min following the uniaxial tensile testing method of the JSCE recommendations [49]. Load was recorded by the machine’s built-in sensor, and displacement was synchronously measured using a TDS-540 high-speed static strain acquisition system. A camera was positioned along the loading direction, fixed on a tripod, and operated with a wireless shutter. The image resolution was 7952 × 5304 pixels, and the lens was approximately 0.4 m from the specimen surface. Images were collected under different illumination conditions to increase dataset diversity. The test and image-acquisition system is shown in Figure 4.

#### 2.1.4. Data Preprocessing and Augmentation

The raw RGB images (7952 × 5304 pixels) acquired during uniaxial tensile tests (conducted at 0.5 mm/min following the same JSCE recommendations [49]) were converted to PNG format and cropped into 256 × 256-pixel patches. Pixel-level ground-truth binary masks were manually annotated using Labelme [50], assigning 255 to crack pixels and 0 to background pixels. In total, 1440 annotated image–mask pairs were collected across 15 dog-bone specimens (Specimens 01–15), with 59 to 133 patches per specimen (mean: 96; individual counts: 88, 97, 90, 98, 77, 59, 104, 104, 68, 68, 118, 105, 105, 126, and 133). Among the 1440 patches, 386 patches (26.8%) contained uncracked matrix surfaces and were retained as negative samples to reduce false-positive predictions on background textures and pores.

**Figure 5:**
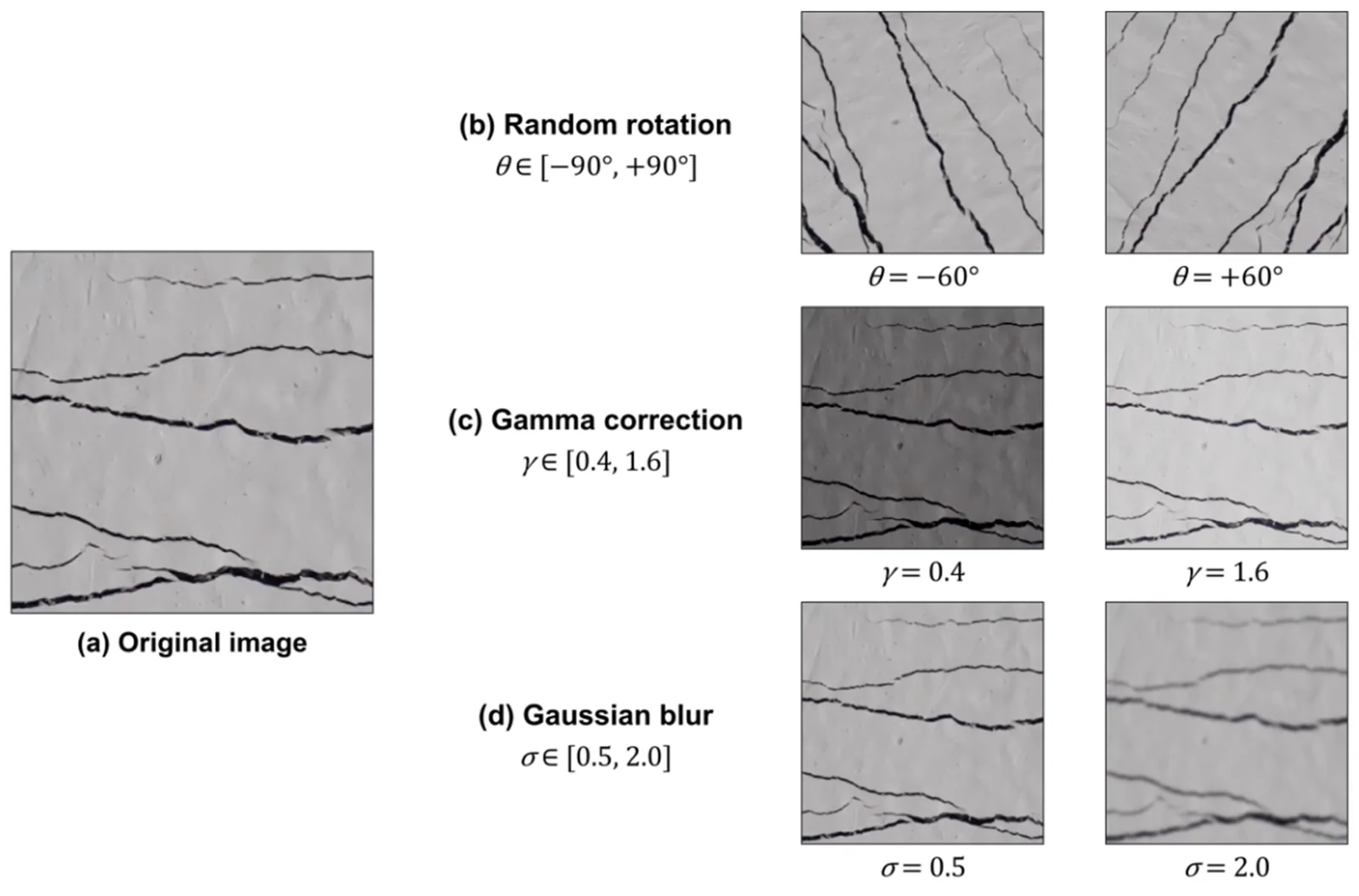
Representative examples of image augmentation.

The 1440 paired patches were randomly partitioned into training (1152 patches, 80%), validation (144 patches, 10%), and test (144 patches, 10%) sets. To ensure reproducibility, four augmented copies were generated from each of the 1152 original training patches, expanding the training set to 5760 images (1152 originals + 4608 augmented variants) [51]. Each augmented copy was produced by sequentially applying three stochastic operations (with application probability *p* = 1.0 per copy): (1) random rotation uniformly sampled from *θ* ∈ [−90°, +90°] using bilinear interpolation for RGB images and nearest-neighbor interpolation for masks, (2) random gamma correction uniformly sampled from *γ* ∈ [0.4, 1.6], and (3) Gaussian blur with kernel standard deviation *σ* ∈ [0.5, 2.0] pixels (Figure 5). The validation and test sets remained strictly unaugmented.

It should be noted that the internal 8:1:1 partition was performed at the image-patch level rather than grouped by physical specimen. While restricting augmentation exclusively to the post-split training subset prevents augmented data leakage, patches originating from the same physical specimen can appear across training and test splits. Because only 15 specimens were available across six mixture proportions, a strict specimen-level 8:1:1 split would leave only one or two specimens in the validation and test sets, rendering evaluation metrics sensitive to individual specimen cracking variability. Consequently, we openly acknowledge that the internal test results reflect patch-level segmentation performance; to rigorously evaluate generalization on completely unseen specimens and imaging setups, independent cross-dataset validations were conducted on the public DeepCrack [42] and Hao-ECC [43] benchmarks (Section 3.1.3).

### 2.2. Overall Architecture of EfficientSegLite

**Figure 6:**
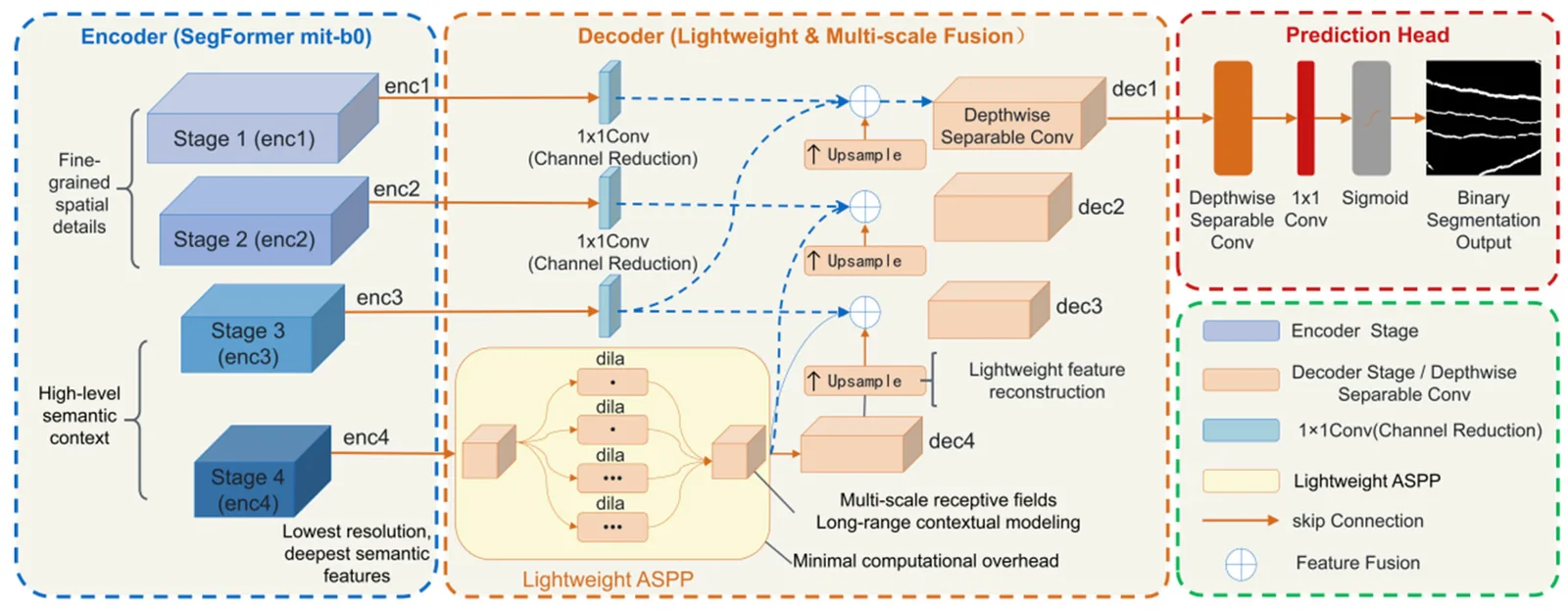
Architecture of the proposed semantic-segmentation model.

EfficientSegLite uses a pretrained SegFormer-B0 encoder to retain spatial detail and semantic context in multiscale features. Channels in the shallow and intermediate features are first compressed using 1 × 1 convolutions and then passed to successive decoder stages through skip connections. At each decoder stage, the upsampled deep features are fused with the corresponding skip features and reconstructed using depthwise separable convolutions [52]. Light-ASPP is applied to the deepest feature stage and uses parallel branches with different dilation rates to incorporate multiscale context. The output head consists of a depthwise separable convolution followed by a 1 × 1 convolution. A sigmoid function produces the pixel-wise probability map. The architecture is illustrated in Figure 6.

The pretrained SegFormer-B0 encoder [53] extracts crack features at four scales through hierarchical self-attention, thereby modeling slender, dispersed cracks that extend across distant regions of an ECC image. For an input image, the encoder outputs four multiscale feature maps, {*E*_1_, …, *E*_4_}. Shallow features retain texture, boundary, and positional information, whereas deeper features provide stronger semantic representations and long-range context. Each feature map is projected through a 1 × 1 convolution to obtain lightweight skip features, {*S_i_*}. This projection standardizes channel dimensions, reduces redundant channels, and lowers the cost of subsequent feature fusion while retaining the details required for crack segmentation. Specifically, the four encoder maps have 32, 64, 160, and 256 channels; the first three are compressed to 16, 32, and 64 channels, and the deepest 256-channel feature supplies the context-processing stage.

Light-ASPP is applied to the deepest feature map, *E*_4_, where Parallel depthwise separable convolution branches with different dilation rates extract multiscale context and enlarge the effective receptive field. This design improves discrimination among cracks of different widths, complex branches, and similar background textures. Compared with conventional ASPP [54], Light-ASPP retains multiscale representation while reducing both parameter count and computation. It contains three parallel 3 × 3 depthwise atrous-convolution branches with dilation rates of 1, 3, and 6, each followed by pointwise channel mixing, together with a global-average-pooling branch.

The decoder follows a top-down multiscale fusion strategy. Deep features are progressively upsampled and fused with skip features at the corresponding scales, combining high-level semantics with shallow spatial detail. OptimizedDWConv reconstructs the fused features at each stage by decoupling spatial feature extraction from channel mixing. Finally, a depthwise separable convolution and a 1 × 1 convolution map the high-resolution feature to a single-channel probability map. Thresholding then produces the binary crack mask.

### 2.3. Composite Optimization Strategy for Fine Cracks

A two-stage loss-weighting strategy was adopted to balance training stability with fine-boundary detection. Stage 1 emphasized Dice loss with a weight of 0.70 and used Focal loss with a weight of 0.30, without edge loss. This setting promoted regional convergence and mitigated class imbalance. Training switched from Stage 1 to Stage 2 adaptively once the validation IoU had plateaued (i.e., no significant improvement over 10 consecutive epochs), with 50 epochs serving only as the maximum cap of Stage 1 (as listed in Table 4).

Stage 2 reduced the Dice-loss weight and increased the Focal- and edge-loss weights to emphasize hard pixels and crack boundaries. Online hard example mining (OHEM) was incorporated into the Focal loss. After warm-up, the OHEM ratio gradually decreased to its target value, focusing optimization on high-loss pixels. This strategy improved segmentation accuracy and boundary consistency while retaining a lightweight architecture.

Dice loss [55] measures the global pixel-wise overlap between the predicted probability *p_i_* and the ground-truth label *y_i_*. A smoothing term, *ε* = 10^−6^, ensures numerical stability for extremely sparse ground truth. The Dice coefficient is calculated for each sample in probability space and then averaged, encouraging agreement in both shape and overall crack-pixel fraction. For a sample containing *N* pixels, Dice loss is defined as follows:

(1) $$L_{\mathrm{Dice}}=1-\frac{2\times \sum_{i}\left(y_{i}^{\mathrm{pred}}\cdot y_{i}^{\mathrm{true}}\right)+\varepsilon }{\sum_{i}y_{i}^{\mathrm{pred}}+\sum_{i}y_{i}^{\mathrm{true}}+\varepsilon }$$

where *p_i_* = *σ*(*z_i_*) is the sigmoid probability of the network logit *z_i_*, *y_i_* ∈ {0, 1} is the ground-truth label, and *ε* = 10^−6^ prevents division by zero.

Focal loss [56] addresses severe class imbalance, while OHEM [57] emphasizes pixels that are difficult to classify during training. This combination prevents numerous easy background pixels from overwhelming narrow cracks and discontinuous boundaries. The pixel-wise Focal loss is expressed as:

(2) $$L_{\mathrm{Focal},i}=-\alpha {y_{i}\left(1-p_{t}\right)}^{\gamma }\log\left(p_{t}\right)-\left(1-\alpha \right)\left(1-y_{t}\right){p_{t}}^{\gamma }\log(1-p_{t})$$

where *α* ∈ [0, 1] is the positive-class weight and *γ* > 0 is the focusing parameter.

The OHEM strategy selects the *k* pixels with the highest losses:

(3) $$L_{\mathrm{Focal}-\mathrm{OHEM}}=\frac{1}{k}\sum_{i\epsilon \mathrm{Top}-k}L_{\mathrm{Focal},i},\;k=\lfloor r\cdot N\rfloor$$

where *r* is the OHEM ratio and *N* is the total number of pixels.

To preserve boundary information explicitly, an edge loss based on Sobel convolution [58] was introduced. Sobel operators are applied to the predicted probability *p* and ground-truth mask *y* to obtain soft edge maps, *E*(*p*), *E*(*y*). Their difference is measured using the L1 mean absolute error. This term directly constrains boundary localization rather than only pixel-wise classification, improving crack continuity and detail reconstruction:

(4) $$E_{x}(p)\;=\;p\;*\;K_{x},\;E_{y}(p)\;=\;p\;*\;K_{y},\;E\left(p\right)=\sqrt{{E_{x}\left(p\right)}^{2}+{E_{y}\left(p\right)}^{2}+\varepsilon }$$

(5) $$E_{x}(y)=y\;*\;K_{x},\;E_{y}(y)=y\;*\;K_{y},\;E\left(y\right)=\sqrt{{E_{x}\left(y\right)}^{2}+{E_{y}\left(y\right)}^{2}+\varepsilon }$$

where *K_x_* and *K_y_* are the Sobel kernels. The symbol “*” denotes convolution. The edge loss is defined using the L1 norm

(6) $$L_{\mathrm{Edge}}=\frac{1}{N}\sum_{i=1}^{N}\left|{E\left(p\right)}_{i}-{E\left(y\right)}_{i}\right|$$

The total loss is expressed as follows:

*L_total_* = *ω_dice_* × *L_Dice_* + *ω_focal_* × *L_Focal_*_−*OHEM*_ + *ω_edge_* × *L_Edge_* (7)

where *ω*_dice_, *ω*_focal_, and *ω*_edge_ denote the weights of the Dice, Focal, and edge losses, respectively.

### 2.4. Crack-Feature Extraction and Quantification

#### 2.4.1. Inference on High-Resolution Images

After training, each test image was read, converted to a tensor, and normalized using the same procedure as the training data. Because the original images had high resolution, a 256 × 256-pixel sliding window was used for patch-wise inference. The predicted patches were reassembled at their original coordinates to obtain a probability map for the complete specimen region.

During full-field inference, each high-resolution gauge-region image was processed via a 256 × 256-pixel sliding window, and patch predictions were reassembled using arithmetic averaging across overlapping regions. The resulting probability map was binarized using a fixed probability threshold of 0.50 without validation-set tuning. Connected components were then labeled [59] and filtered using morphological criteria—requiring component area ≥ 200 pixels, aspect ratio ≥ 1.0, eccentricity ≥ 0.1, maximum width ≤ 2.0 mm, and circularity ≤ 0.8—to eliminate false positives induced by surface pores, rubber particles, and illumination noise. The retained regions were skeletonized to obtain one-pixel-wide centerlines while preserving topology [60]. Median filtering was used only to smooth visualizations [58]. Masks used to calculate width and area were not dilated because dilation would alter the physical dimensions.

Because excessive area filtering can remove genuine nascent microcracks, the physical scale and sensitivity of the 200-pixel threshold were evaluated. Given the calibrated physical resolution of *k_x_* = 0.0170 mm/px and *k_y_* = 0.0167 mm/px (~2.84 × 10^−4^ mm^2^/px), an area of 200 pixels corresponds to an effective physical area of ~0.057 mm^2^ (equivalent to a fine microcrack segment ~50 μm wide and ~1.1 mm long). Sensitivity evaluation across area thresholds of 100, 150, 200, 250, and 300 pixels on the six full-field binary masks showed that, between any two thresholds in this range, the extracted total crack area and network mean width varied by less than 3% (maximum deviations of +2.6% and −1.7%, respectively). Thresholds below 100 pixels (~0.028 mm^2^) admitted numerous sub-millimeter surface pores as spurious cracks, whereas thresholds above 300 pixels (~0.085 mm^2^) began discarding short, isolated microcracks during early crack initiation. Thus, 200 pixels provides a practical trade-off between pore-noise suppression and microcrack retention, although we acknowledge that isolated nascent microcracks smaller than 0.057 mm^2^ may be filtered out.

#### 2.4.2. Crack Morphology and Scale Conversion

Connected crack regions were labeled from binary segmentation and small-scale noise was removed using an area threshold. Total crack area and crack area density within the observed region were then calculated. Circularity, aspect ratio, and Euler number [59] were additionally extracted to describe crack morphology, extension, and connectivity.

#### 2.4.3. Skeleton-Based Calculation of Crack Length and Width

The processed binary masks were used to calculate crack area, area density, length, width, and morphological complexity. Total crack area was obtained by summing the physical area represented by all crack pixels. Crack area density was defined as the ratio of total crack area to the observed area. Aspect ratio, Euler number, and branch-node count characterized crack extension, connectivity, and topological complexity.

Crack length was calculated from a one-pixel-wide skeleton [60] to reduce the effects of width variation and local boundary irregularity. With eight-neighbor connectivity, adjacent skeleton pixels form horizontal or vertical links and diagonal links. A direct link corresponds to one pixel unit, whereas a diagonal link corresponds to $\sqrt{2}$ pixel units. The total crack length in pixels is therefore:

(8) $$L_{\mathrm{px}}=\frac{1}{2}(N_{\mathrm{straight}}+\sqrt{2}N_{\mathrm{diagonal}})$$

where *N*_straight_ and *N*_diagonal_ are the numbers of direct and diagonal skeleton links, respectively. The coefficient 1/2 corrects for double counting of adjacent pixel pairs.

For crack-width calculation, the crack boundary was extracted from the binary mask and the skeleton was treated as the crack centerline. For any skeleton point *p* ∈ Ω*_s_*, the local crack width was defined as twice the physical distance from that point to the nearest boundary point using the Euclidean distance transform [61], as given in Equation (9):

(9) w(p)=2‖p−q‖2qϵΩemin

Here, the skeleton-point set and boundary-point set are denoted by Ω*_s_* and Ω*_e_*, respectively. Because the horizontal and vertical physical resolutions can differ, the physical distance between skeleton point *p* and boundary point *q* is expressed as:

(10) $${\left\|p-q\right\|}_{2}=\sqrt{{\left(\Delta x\cdot s_{x}\right)}^{2}+{\left(\Delta y\cdot s_{y}\right)}^{2}}$$

where Δ*x* and Δ*y* are the coordinate differences between the skeleton and boundary points. The quantities *s_x_* and *s_y_* are the horizontal and vertical physical resolutions, respectively. This conversion maps pixel-scale distances into physical dimensions and maintains dimensional consistency under anisotropic imaging resolution.

Coupling crack segmentation with physical calibration is an established measurement approach [39,40]. A mapping between image coordinates and physical dimensions was established to convert ECC crack measurements from pixels to millimeters. Separate scale factors were used so that the reported geometric quantities retained clear physical meaning.

Calibration was based on the known specimen geometry. Let the physical width and height of the gauge region be *W_mm_* and *H_mm_*, respectively. The corresponding pixel width and height, determined by manual interaction or a predefined crop, are *W_px_* and *H_px_*. The scale factors in the x and y directions, *k_x_* and *k_y_*, are defined as:

(11) $$k_{x}=\frac{W_{\mathrm{mm}}}{W_{\mathrm{px}}},\;k_{y}=\frac{H_{\mathrm{mm}}}{H_{\mathrm{px}}}$$

where *k_x_* and *k_y_* are expressed in millimeters per pixel (mm/px). Separate scale factors accommodate possible image-scale distortion and preserve geometric fidelity.

During uniaxial tensile testing, the camera was mounted on a rigid tripod approximately 0.4 m from the specimen with its optical axis aligned perpendicular to the flat specimen surface (Figure 4). The central 30 mm × 80 mm gauge region (*W_mm_* = 30 mm, *H_mm_* = 80 mm) was bounded by the upper and lower LVDT mounting frames and extracted via a consistent region-of-interest (ROI) crop of *W_px_* = 1760 pixels by *H_px_* = 4800 pixels, yielding horizontal and vertical scale factors of *k_x_* = 0.0170 mm/px and *k_y_* = 0.0167 mm/px (Equation (11)). Under this controlled, near-orthographic planar laboratory configuration, perspective foreshortening across the flat 30 mm × 80 mm gauge zone is negligible. Nevertheless, because intrinsic lens distortion parameters and homography matrices were not calibrated, extending this workflow to handheld or oblique field inspections of curved or large-scale structures will require checkerboard camera calibration and perspective rectification.

### 2.5. Intelligent Inspection System

**Figure 7:**
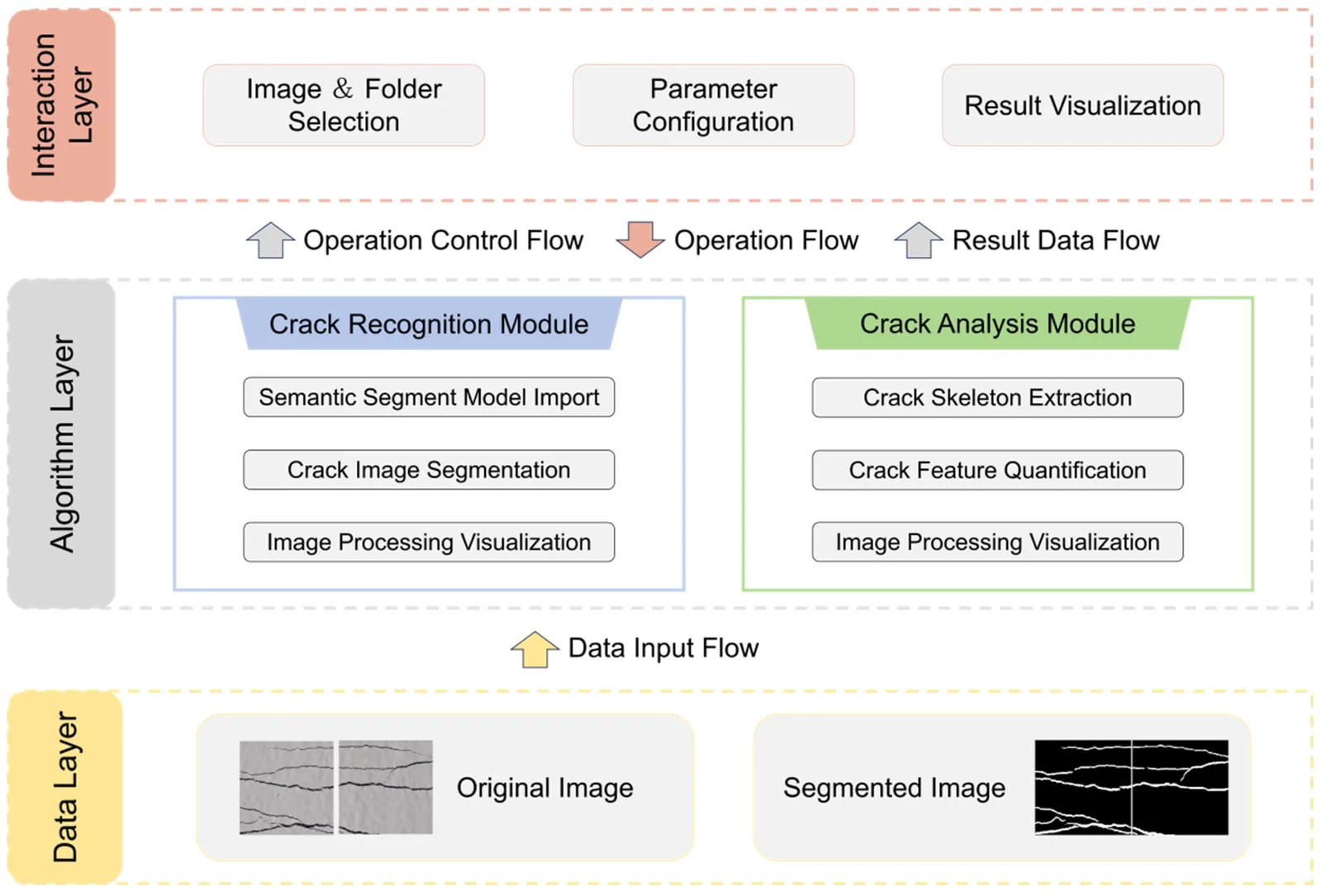
Architecture of the intelligent crack inspection and analysis system.

An intelligent ECC crack inspection and analysis system was developed to integrate crack segmentation with geometric quantification (Figure 7). The system receives raw images and outputs crack masks, geometric parameters, and structured analytical text. Its workflow includes image preprocessing, semantic segmentation, feature extraction, scale conversion, and result interpretation.

The system consists of a local crack-detection and quantification platform and a tool-calling LLM agent. The local platform performs image processing, model inference, and geometric calculations. The agent parses natural-language instructions, invokes tools, organizes results, and generates text. Both components use the same EfficientSegLite model and crack-quantification algorithms and exchange images, masks, skeletons, and quantitative parameters through a unified interface.

#### 2.5.1. Crack-Detection and Quantification Platform

The local platform was developed in PyQt5 and comprises data-processing, algorithmic-computation, and interaction-management layers. The layers communicate through a predefined data flow that integrates image loading, crack segmentation, and parameter calculation.

The data-processing layer accepts either a single image or an image folder and performs format validation, region-of-interest cropping, size standardization, tensor conversion, and pixel normalization. High-resolution ECC images are divided into patches using a predefined window, and their original coordinates are recorded for subsequent reconstruction.

The algorithmic-computation layer includes crack-segmentation and feature-quantification modules. The segmentation module uses EfficientSegLite for pixel-wise prediction and converts the probability map into a binary mask. Patch-level predictions are reassembled at their original coordinates to recover the complete crack distribution in the observed region.

The feature-quantification module sequentially performs connected-component filtering, skeletonization, boundary extraction, and branch-node detection. The pixel-to-physical scale mapping is then used to calculate crack length, local width, mean width, area, area density, tortuosity, and fractal dimension. A unified data format allows the modules to be executed sequentially.

The interaction-management layer controls model configuration, image input, task execution, process monitoring, and result storage but does not participate in crack detection or numerical calculation. The interface and computation threads operate independently to prevent inference and geometric calculations from blocking user interaction.

#### 2.5.2. Large Language Model Agent

A tool-calling LLM agent was built on the crack-segmentation and geometric-quantification modules. The system uses the DeepSeek API to parse text instructions and uploaded images and to organize the corresponding analytical workflow. A Streamlit interface supports image upload, natural-language input, result display, and report export.

The system first checks whether an image has been supplied. If an image is present, the agent invokes the crack-segmentation and feature-quantification programs to detect cracks, extract parameters, and prepare analytical text. All numerical values, including crack width, length, area density, and fractal dimension, are calculated by deterministic algorithms. The LLM only interprets the request, organizes the workflow, and expresses the results. Without an image, the system enters a text-only question-answering mode for queries concerning ECC materials, crack characteristics, and analytical indicators.

This workflow connects natural-language interaction, the LLM API, and the crack-analysis programs. Users can trigger a task by uploading an image or entering text, enabling continuous processing from image input and crack detection to parameter quantification, result interpretation, and report generation.

### 2.6. Experimental Settings

The model was trained end-to-end for 100 epochs under supervised learning. The training phases and loss-function settings are summarized in Table 4 and Table 5. Training and performance evaluation were conducted in Python 3.11 using PyTorch 2.4.1. The workstation contained an NVIDIA GeForce RTX 3060 Ti GPU and an Intel Core i5-13400F CPU, and CUDA 12.4 was used for acceleration.

AdamW [62] was used for optimization. The encoder learning rate was 1 × 10^−4^, while the decoder and prediction-head learning rates were 1 × 10^−3^. Weight decay was 1 × 10^−4^. ReduceLROnPlateau [63] adjusted the learning rate according to validation IoU. The reduction factor was 0.8, patience was 20 epochs, and the minimum learning rate was 1 × 10^−5^. Training and validation IoU were recorded after each epoch. The complete settings are listed in Table 6.

#### 2.6.1. Semantic-Segmentation Metrics

Six metrics were used to evaluate ECC crack segmentation: pixel accuracy (PA), mean pixel accuracy (MPA), mean intersection over union (mIoU), recall, precision, and F1 score.

All evaluation metrics were derived from the confusion matrix. *P_ii_* denotes pixels whose true class *i* is predicted as class *i*. Depending on the class, these entries represent true positives (TP) or true negatives (TN). *P_ij_* denotes pixels whose true class is *i* but predicted class is *j*, corresponding to false positives (FPs) or false negatives (FNs). If class *i* is the positive class and *i* ≠ *j*, then *P_ii_*, *P_jj_*, *P_ji_*, and *P_ij_* represent TP, TN, FP, and FN, respectively.

The evaluation metrics were calculated as follows:

(12) $$\mathrm{PA}=\frac{\mathrm{TP}+\mathrm{TN}}{\mathrm{TP}+\mathrm{FP}+\mathrm{FN}+\mathrm{TN}}$$

(13) $$\mathrm{MPA}=\frac{\sum_{i=1}^{m}\mathrm{PA}}{m}$$

(14) $$\mathrm{mIoU}=\frac{\frac{\mathrm{TP}}{\mathrm{TP}+\mathrm{FP}+\mathrm{FN}}+\frac{\mathrm{TN}}{\mathrm{FP}+\mathrm{FN}+\mathrm{TN}}}{2}$$

(15) $$\mathrm{precision}=\frac{\mathrm{TP}}{\mathrm{TP}+\mathrm{FP}}$$

(16) $$\mathrm{recall}=\frac{\mathrm{TP}}{\mathrm{TP}+\mathrm{FN}}$$

(17) $$F1\;\mathrm{score}=\frac{2\mathrm{TP}}{2\mathrm{TP}+\mathrm{FP}+\mathrm{FN}}$$

#### 2.6.2. Crack-Characterization Metrics

A set of geometric metrics was derived from the segmentation results to characterize multiple cracking in ECC. These metrics comprised crack area, total length, mean width, tortuosity, fractal dimension, and visual equivalent strain. Their definitions are provided in Table 7.

(1) Crack area. Crack area was defined as the total physical area represented by all crack pixels in the segmented image. It describes the overall extent of cracking during tension and incorporates crack number, length, and width. It therefore provides a more comprehensive measure of distributed multiple-cracking damage than either a single crack width or crack count.
(2) Total crack length. Total crack length was defined as the summed physical length of all skeleton pixels in an image. A medial-axis transform extracted the crack skeleton, reducing interference from crack-width variation. This metric describes the scale of the surface crack network and provides a geometric indicator of multiple-crack development and crack saturation in ECC:

(18) $$\Sigma L_{\mathrm{skeleton}}=\sum_{i=1}^{n}L_{i}$$

where *L_i_* is the skeleton length of the *i*th crack, in mm.

(3) Mean crack width. During strain hardening, ECC develops complex crack branches, intersections, and overlaps. Conventional single-point measurements are therefore sensitive to local noise and geometric variation. An area-to-length ratio was used to define an equivalent mean crack width:

(19) $$\bar{w}=\frac{A_{C}}{\Sigma L_{\mathrm{skeleton}}}$$

where *A_C_* is the total crack area and Σ*L_skeleton_* is the total skeleton-based crack length.

This definition represents the statistical opening of the crack network and reduces sensitivity to local measurement noise. It is particularly suitable for dense networks of narrow, highly branched cracks in ECC.

(4) Crack tortuosity (*τ*). Tortuosity describes the curvature of a crack path and quantifies crack-propagation complexity. It is defined as the ratio of the actual skeleton-based path length to the straight-line distance between the two crack endpoints:

(20) $$\tau =\frac{L_{\mathrm{act}}}{L_{\mathrm{str}}}$$

where *L_act_* is the actual crack length and *L_str_* is the Euclidean distance between its endpoints. A higher tortuosity indicates a more winding surface path. Such two-dimensional geometric observations are consistent with crack-deflection mechanisms induced by compliant rubber inclusions and bridging fibers reported in the literature [17,64,65,66], although image-derived tortuosity alone does not isolate their individual micromechanical contributions.

(5) Fractal dimension (*D_f_*). Fractal dimension describes the spatial complexity and self-similarity of a crack network and was calculated using the box-counting method [67]. It reflects network density and geometric roughness:

(21) $$D_{f}=\underset{\delta \to 0}{\lim}\frac{\log N(\delta )}{\log\left(1/\delta \right)}$$

where *δ* is the box side length and *N*(*δ*) is the number of boxes containing crack pixels. A higher fractal dimension indicates a denser and more developed crack network and provides a measure of the spatial uniformity of distributed damage.

(6) Crack-based visual equivalent strain. A visual equivalent strain was derived from total crack area to estimate macroscopic strain. Assuming that crack opening displacement is uniformly distributed across the width of the gauge region, the visual equivalent strain is defined as:

(22) $$\varepsilon_{v}=\frac{A_{C}}{B\cdot L_{g}}$$

where *A_C_* is the total crack area, *B* is the width of the specimen gauge region, and *L_g_* is the strain-measurement gauge length. Geometrically, this metric represents the cumulative contribution of all crack openings in the loading direction. It allows the evolution of macroscopic strain during multiple cracking to be quantified without a physical displacement sensor. The expression is a two-dimensional crack-area fraction: it assumes that cracks are approximately transverse to the loading direction, extend across the gauge width, and open uniformly, and it does not include deformation of the uncracked matrix or unresolved subpixel crack openings. Its use as a quantitative strain proxy therefore requires mixture-specific calibration and agreement assessment.

## 3. Results and Discussion

This section evaluates the crack-segmentation performance and engineering applicability of EfficientSegLite. Section 3.1 compares models and training strategies and uses ablation experiments to isolate the contributions of key components. Section 3.2 quantifies crack geometry and evaluates measurement accuracy and correlation with measured strain. Section 3.3 demonstrates integration of the model into the intelligent inspection system.

### 3.1. Model Performance

#### 3.1.1. Training Results

**Figure 8:**
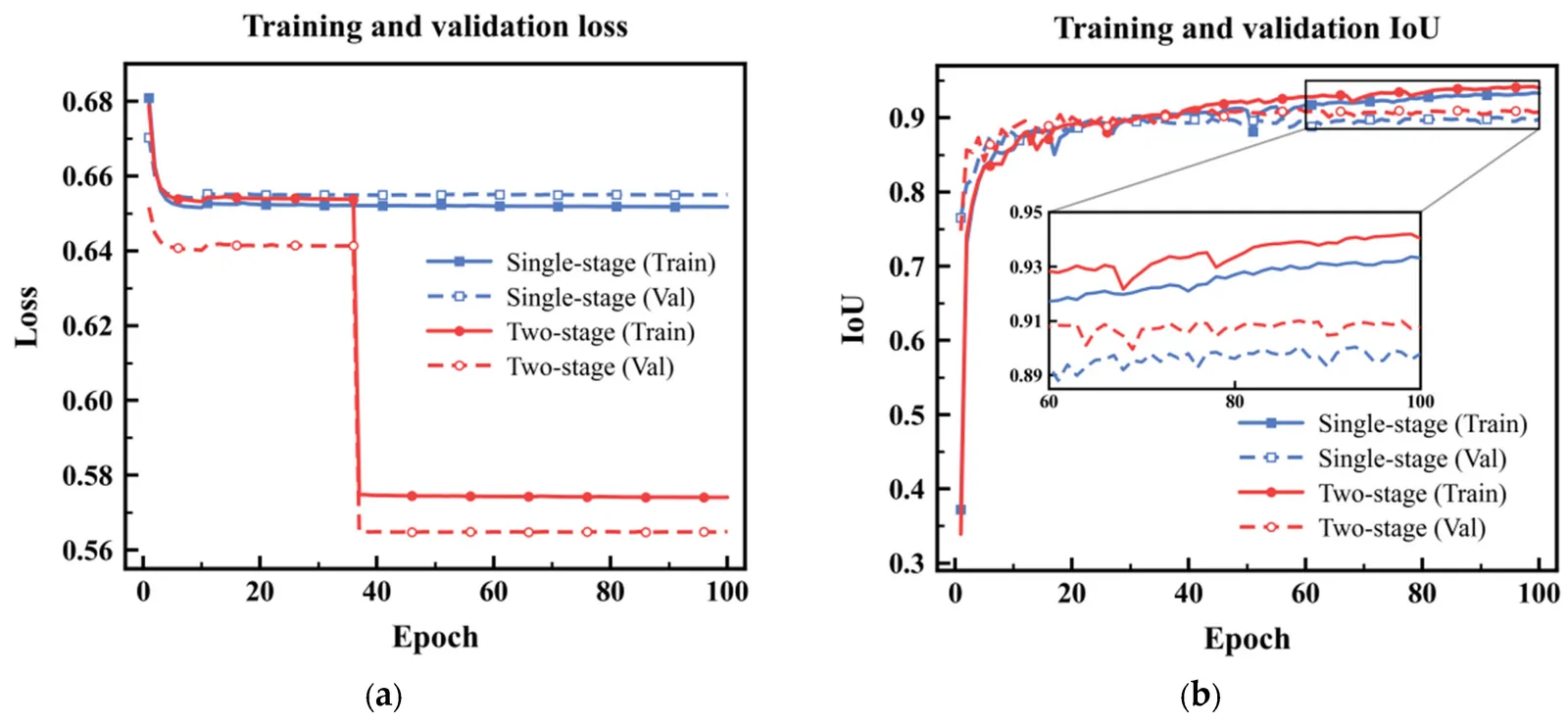
Training results: (**a**) loss curves; (**b**) IoU curves.

Figure 8 compares the loss and IoU curves obtained using single-stage and two-stage training. Reweighting the loss terms at the start of Stage 2—triggered at approximately epoch 36 under this validation-IoU convergence criterion—produced a step change in the total training and validation losses. This change reflected a rescaling of the optimization objective rather than training instability. IoU is unaffected by loss-weight scaling and is therefore more appropriate for evaluating segmentation. The two strategies produced similar early IoU trajectories. After Stage 2 began, the validation mIoU of the two-stage strategy continued to increase and stabilized at approximately 0.934. These results indicate that OHEM directed the model toward fine boundaries and hard pixels and improved precision and training stability.

The repeated experiments in Table 8 show that the two-stage strategy maintained high mean pixel accuracy. It achieved an mIoU of 93.39 ± 0.04% and an F1 score of 93.24 ± 0.04%. Although the single-stage strategy produced slightly higher recall, its precision was only 90.19% and varied substantially (±2.69%). This pattern indicates that the single-stage model more often misclassified matrix texture as cracks. The two-stage strategy increased precision to 93.47% with an SD of 0.55%, substantially reducing false positives. Its mIoU SD was 0.04%, compared with 0.63% for single-stage training, demonstrating more stable convergence across repeated runs.

To evaluate the statistical significance of the differences between single-stage and two-stage training across the three repeated runs (*n* = 3, Table 8), two-sided Welch’s t-tests on the sample means and F-tests on the run-to-run variances were conducted. Compared with single-stage training, the two-stage strategy improved mean mIoU from 92.81% to 93.39% (+0.58%, *t* = 1.59, *p* = 0.252), mean Precision from 90.19% to 93.47% (+3.28%, *t* = 2.07, *p* = 0.165), and mean F1 score from 92.54% to 93.24% (+0.70%, *t* = 1.48, *p* = 0.277). We candidly note that owing to the small number of repeated runs (*n* = 3) and the large run-to-run variance of the single-stage baseline, these differences in mean values do not reach statistical significance at the *α* = 0.05 level. However, F-tests on the sample variances reveal that the two-stage strategy achieved a statistically significant reduction in run-to-run variability for mIoU (SD reduced from 0.63% to 0.04%, F = 248.1, *p* = 0.0040), F1 score (SD reduced from 0.82% to 0.04%, F = 420.2, *p* = 0.0024), and Precision (SD reduced from 2.69% to 0.55%, F = 23.9, *p* = 0.0401). These results demonstrate that the principal advantage of the two-stage Dice–OHEM–Edge schedule lies in significantly stabilizing convergence and suppressing false-positive fluctuations caused by background matrix textures.

#### 3.1.2. Comparison with Baseline Networks

EfficientSegLite was compared with U-Net [68], SegFormer-B0/B1/B2 [53], a SegFormer-B2 + U-Net hybrid [53,68], DeepLabV3+ [54], DDRNet [69], and BiSeNet V2 [70] on the in-house ECC crack-image dataset. The results are presented in Table 9.

EfficientSegLite achieved an MPA of 96.78% and an mIoU of 93.40%, both higher than those of the listed baselines. Its recall was 94.01%, matching the highest value obtained by DeepLabV3+, while its precision and F1 score were 92.85% and 93.24%, respectively. The model required 734.9 M FLOPs and 3.96 M parameters, lower than most high-accuracy baselines. These results show that EfficientSegLite balanced crack detection, false-positive control, and computational efficiency.

#### 3.1.3. Cross-Domain Validation

**Figure 9:**
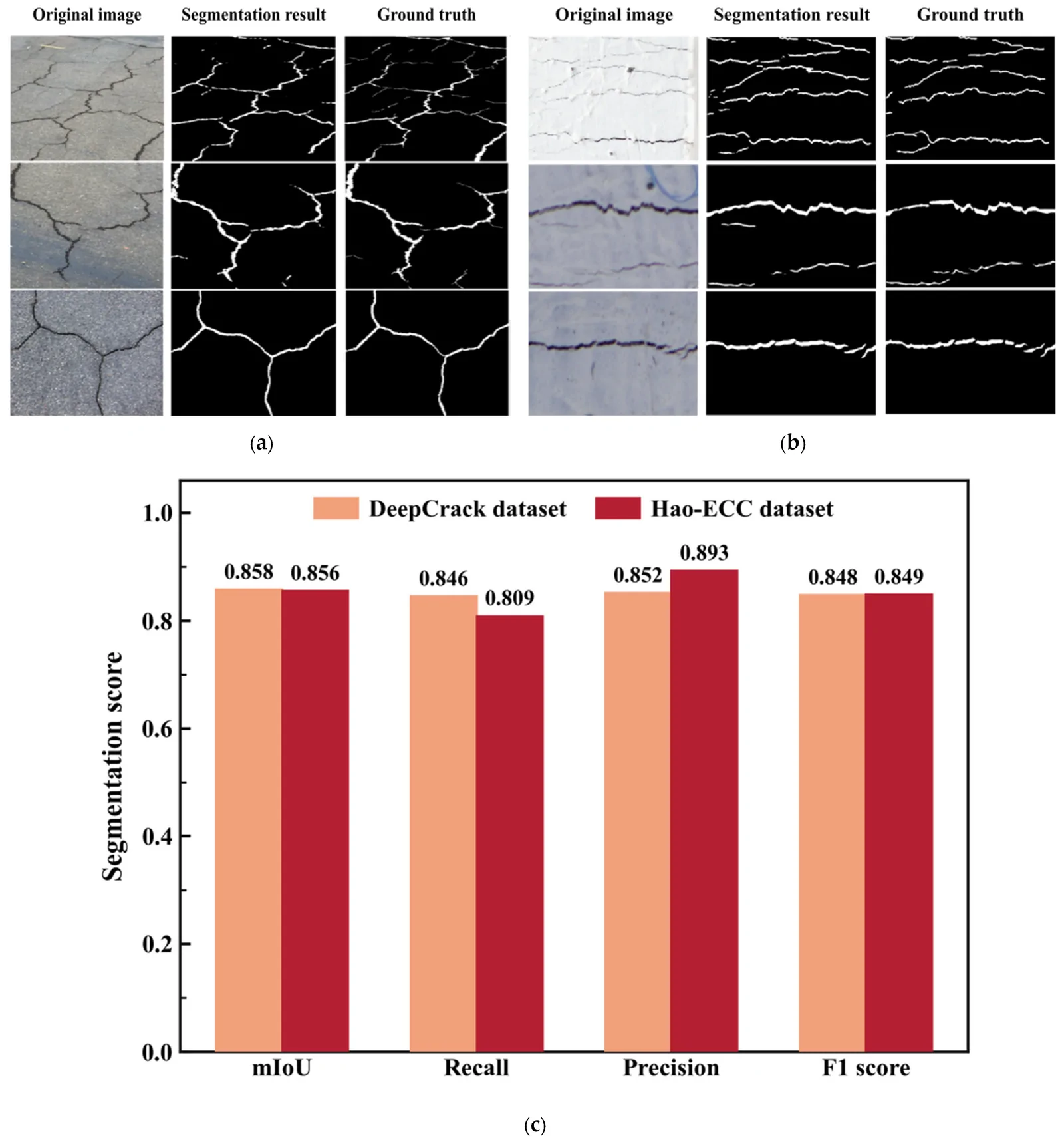
Cross-domain performance on two public crack datasets: (**a**) representative segmentation results on DeepCrack; (**b**) representative segmentation results on the Hao-ECC dataset; (**c**) comparison of mIoU, recall, precision, and F1 score.

To rigorously assess generalization beyond the internal specimens, cross-domain validation was performed on two public benchmarks with distinct material characteristics, spatial resolutions, and imaging conditions: DeepCrack [42] and the Hao-ECC dataset [43] (Figure 9). DeepCrack consists of 537 manually annotated RGB images (544 × 384 pixels) captured across diverse field concrete and asphalt pavement surfaces [42]. Its cracks are predominantly single or sparsely branched macrocracks embedded in rough aggregate textures, shadows, and surface stains, representing a substantial domain shift in both material type and background complexity. Conversely, the Hao-ECC dataset comprises 604 annotated image–mask pairs (480 × 352-pixel patches cropped from 4016 × 6016-pixel raw photographs, covering a ~28 × 42 mm field of view at ~7 μm/px) of dense multiple microcracks on tensile-tested ECC specimens [43], representing an independent cross-laboratory and cross-scale benchmark within the ECC domain.

As shown in Figure 9c, EfficientSegLite achieved mIoU values of 0.858 and 0.856, recall values of 0.846 and 0.809, precision values of 0.852 and 0.893, and F1 score of 0.848 and 0.849 on DeepCrack and Hao-ECC, respectively. The balanced recall and precision on DeepCrack confirm that the multiscale encoder–decoder structure transfers effectively to wider concrete/asphalt macrocracks despite complex background clutter. On Hao-ECC, the higher precision (0.893) paired with lower recall (0.809) reflects a conservative detection profile when transferring from our ~17 μm/px training scale to ultrafine microcracks captured at ~7 μm/px. Together, these external evaluations confirm robust cross-specimen and cross-domain transferability.

#### 3.1.4. Ablation Study

**Figure 10:**
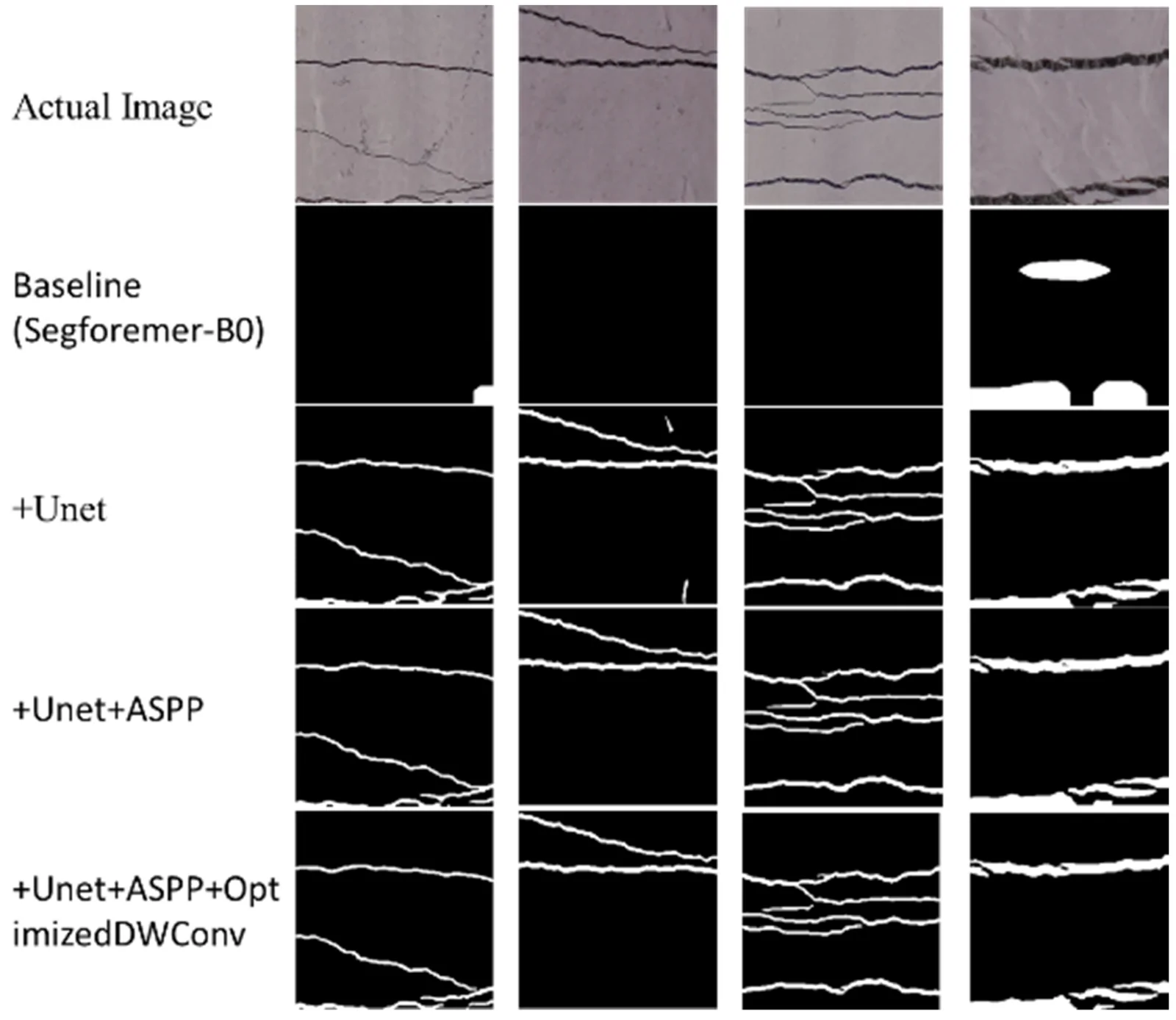
Qualitative comparison of the ablation experiments.

Ablation experiments were conducted using SegFormer-B0 as the baseline, as summarized in Table 10 and Figure 10. The baseline missed cracks and produced discontinuous predictions. Adding U-Net skip connections improved continuity and increased the mIoU and F1 score to 92.76% and 92.33%, respectively. ASPP further reduced background false positives but increased the parameter count. Replacing the conventional convolution blocks with OptimizedDWConv produced an mIoU of 93.40% and an F1 score of 93.24% while reducing both parameter count and FLOPs.

### 3.2. Crack Characterization and Analysis

#### 3.2.1. Measurement Accuracy and Error Analysis

**Figure 11:**
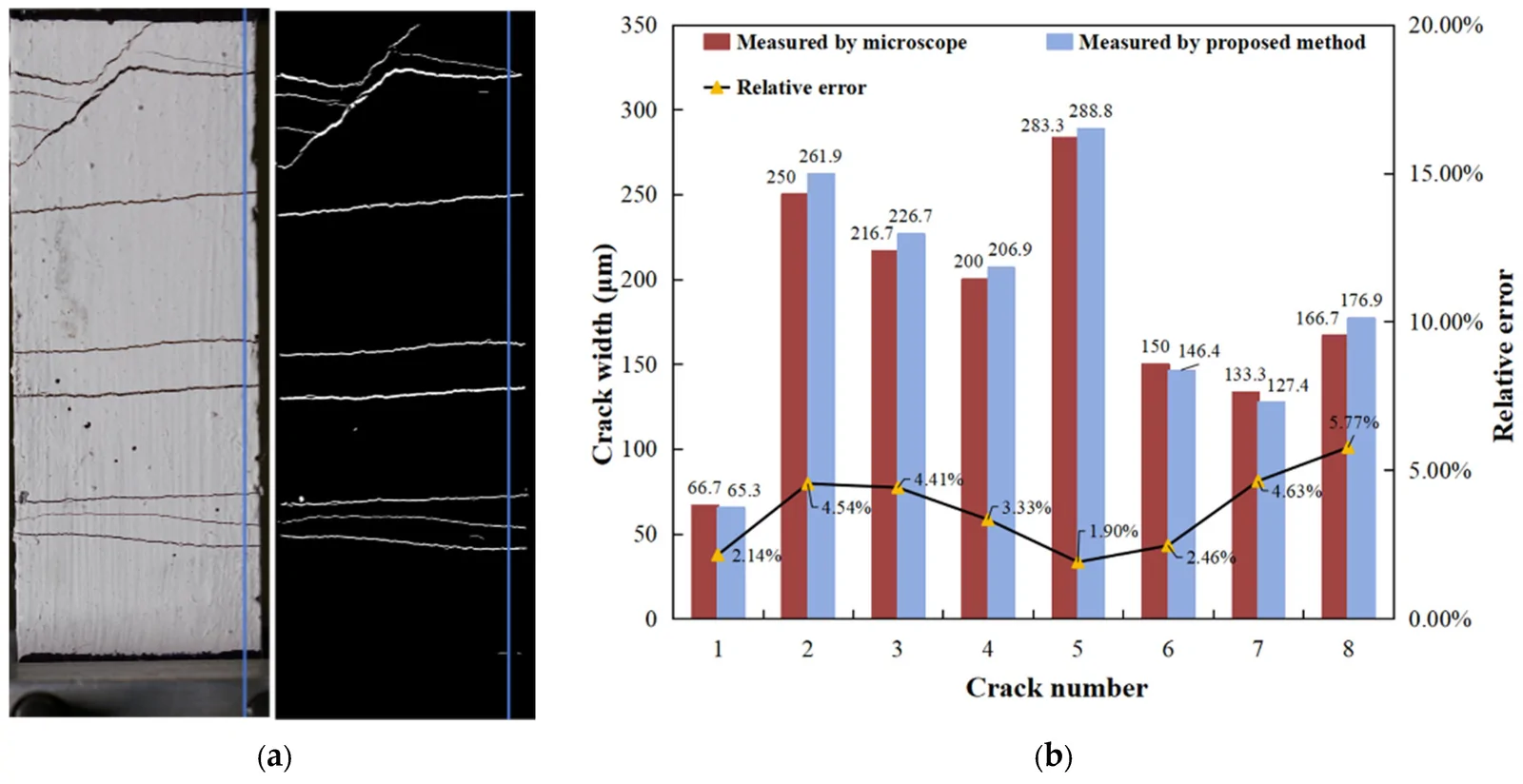
Crack widths measured by optical microscopy and by the proposed method: (**a**) locations of crack-width measurements; (**b**) comparison of crack widths and relative errors. The blue lines indicate the locations selected for crack-width measurement.

Optical microscopy measurements were used to benchmark the physical accuracy of the skeleton-and-EDT crack-width algorithm. Eight representative crack locations along the tensile specimen in Figure 11a (spanning crack widths from 66.7 μm to 283.3 μm) were measured by optical microscopy and compared against the automated image-based estimates (Figure 11b). Across all eight benchmark cracks, the automated measurements agreed closely with the microscopic readings, yielding relative errors between 1.90% and 5.77% (mean relative error: 3.65%).

While this comparison confirms high measurement accuracy for well-resolved microcracks within the 65–290 μm range, we candidly acknowledge the limited sample size (*n* = 8) as an experimental limitation. Because microscopic readings had to be manually acquired during brief loading holds, only eight target points were recorded. Furthermore, pixel-based width estimation is inherently constrained by the physical pixel scale (~17 μm/px), segmentation boundary precision, and skeleton topology at dense crack intersections. Consequently, relative errors are expected to increase for subpixel microcracks (<30 μm) near the optical resolution limit, and broader paired microscopic datasets across multiple loading stages are warranted in future investigations.

#### 3.2.2. Trends in Crack Characteristics

**Figure 12:**
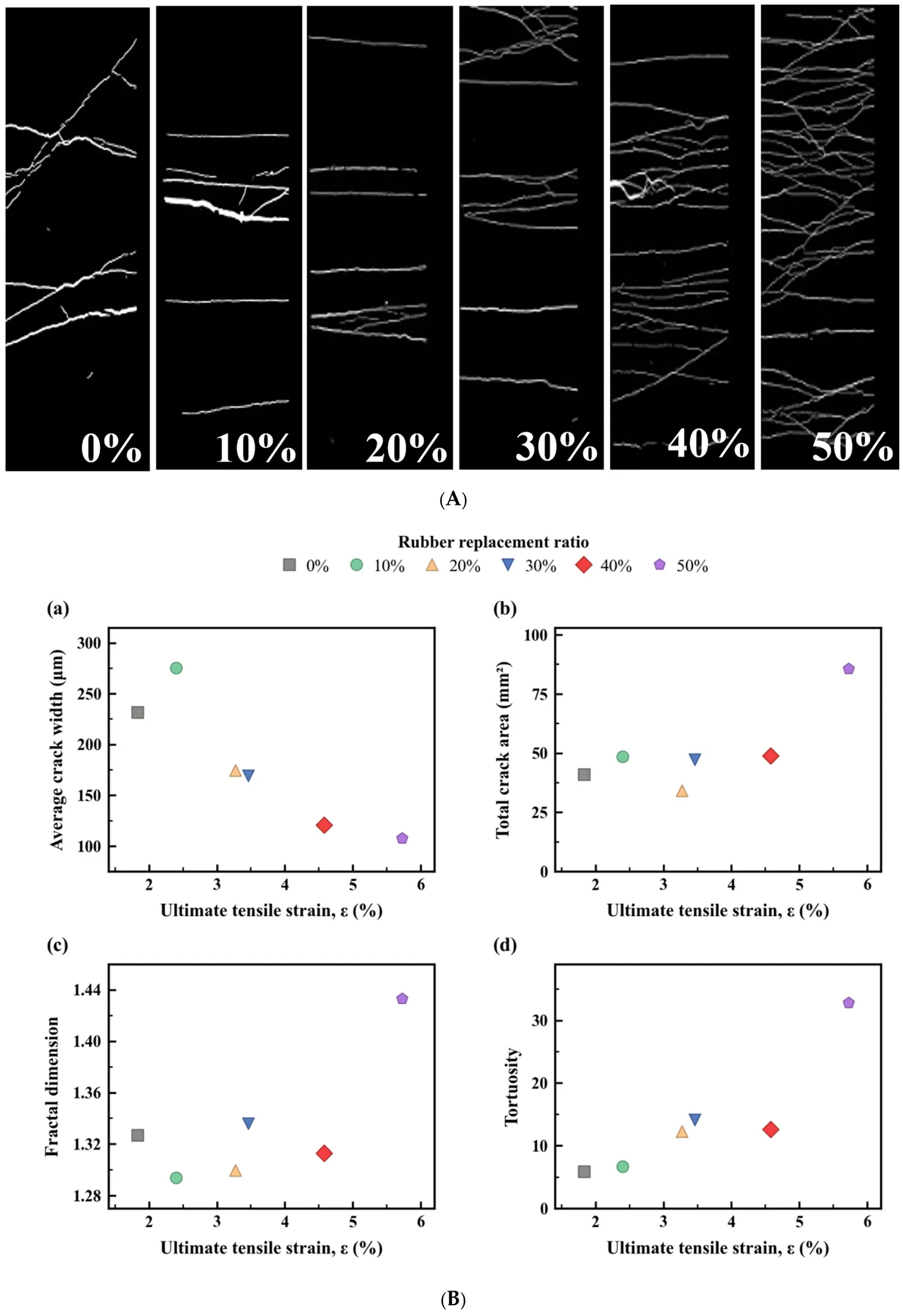
Quantitative crack analysis at different rubber replacement ratios: (**A**) detection results; (**B**) crack parameters varied with ultimate tensile strain and rubber content (**a**–**d**).

The segmentation and parameter-extraction workflow was applied across rubberized ECC specimens with volumetric rubber replacement ratios increasing from 0% to 50% (Figure 12). As the rubber content increased from 0% to 50%, the surface failure pattern transitioned markedly from a limited number of wider cracks to a dense, saturated network of fine microcracks (Figure 12A). Quantitatively, the network mean crack width decreased by 56.3%, from approximately 245 μm (at 0% rubber) to 107 μm (at 50% rubber), whereas the total crack area, crack tortuosity (*τ*), and fractal dimension (*D_f_*) increased progressively with ultimate tensile strain and rubber content (Figure 12B).

These quantitative morphological trends are consistent with established micromechanical principles of rubberized ECC reported in the literature [17,64,65,66]. According to pseudo-strain-hardening theory, steady-state multiple cracking requires both the matrix cracking strength to remain below the maximum fiber-bridging stress (strength criterion) and the matrix fracture toughness (*J*_tip_) to remain below the complementary energy of fiber bridging (energy criterion) [1,64]. Because powdered rubber particles possess an elastic modulus orders of magnitude lower than that of the surrounding quartz–cement matrix and form weak interfacial transition zones, they function as distributed artificial flaws and local stress concentrators [64,65]. Increasing the rubber replacement ratio systematically reduces *J*_tip_ and the matrix cracking stress, thereby activating a substantially larger number of microcracks at smaller load increments while restricting the opening displacement of individual cracks [65,66]. Simultaneously, as propagating microcracks encounter compliant rubber inclusions and bridging PE fibers, the crack tips undergo local stress-field perturbation, pinning, and path deflection rather than propagating along a straight cleavage plane [17,65]. At the macroscopic specimen surface, this repeated deflection and secondary branching manifest as higher skeleton tortuosity (*τ*) and a higher box-counting fractal dimension (*D_f_*). It should be noted that while our 2D optical metrics quantitatively capture these surface geometric signatures, direct visualization of sub-surface crack–rubber–fiber interactions requires complementary microstructural techniques such as scanning electron microscopy (SEM) or X-ray computed tomography (X-CT).

#### 3.2.3. Crack-Width Distribution

**Figure 13:**
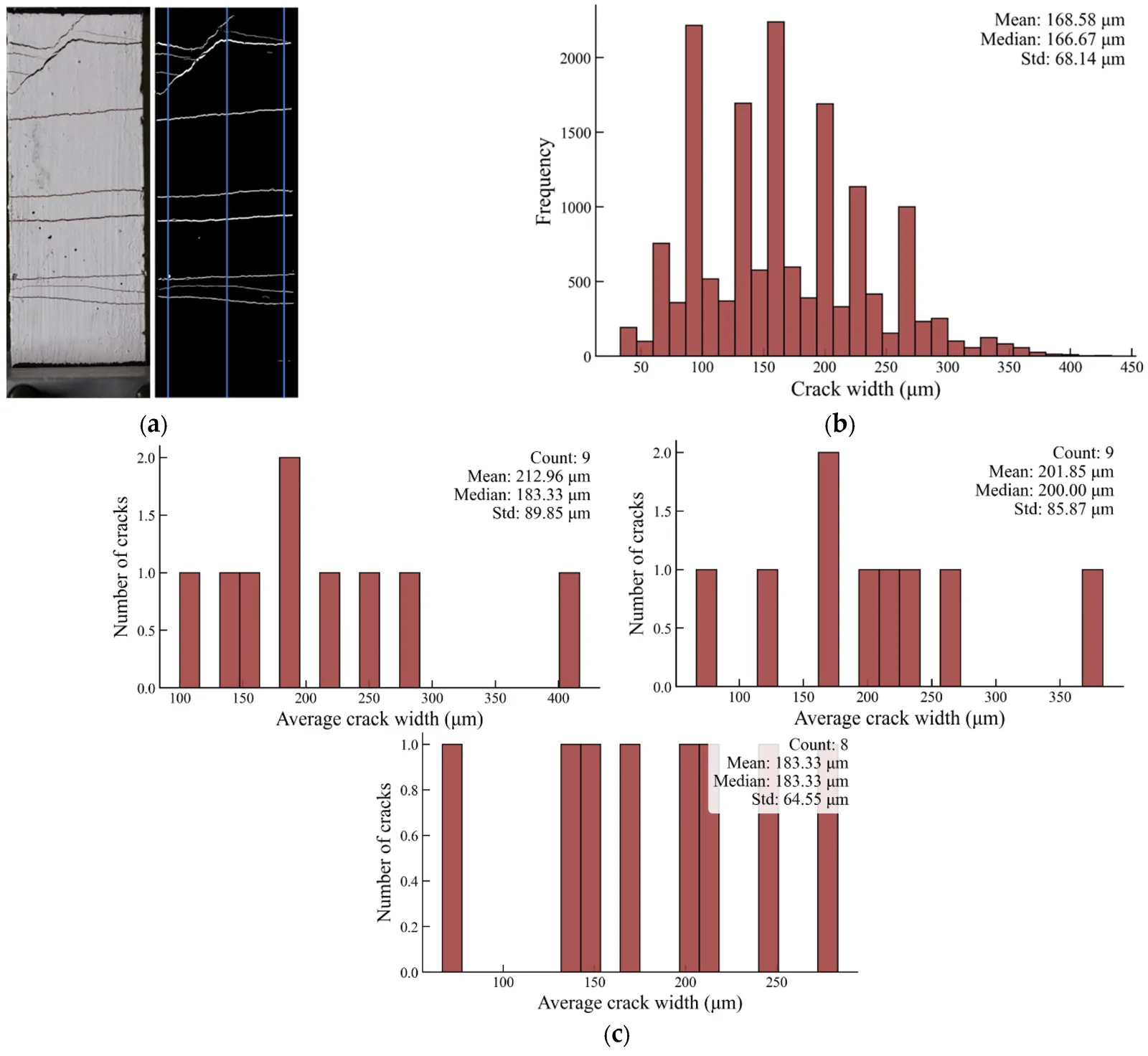
Analysis of crack-width distributions: (**a**) positions of vertical sampling lines; (**b**) global crack-width distribution; (**c**) local crack-width distributions.

Semantic segmentation was further used to quantify local damage during ECC tensile testing, as shown in Figure 13. The extracted crack widths were concentrated between 100 and 250 μm, consistent with the expected stable development of microcracks during strain hardening. The global mean width of 168.58 μm was close to the median of 166.67 μm. The small difference indicates that a limited number of extreme values did not substantially shift the center of the distribution.

Local sampling lines also captured spatial heterogeneity in the ECC crack distribution. Depending on position, the number of intersected cracks ranged from eight to nine, while the local standard deviation decreased from 89.85 μm to 64.55 μm. This spatial variation is consistent with stochastic cracking under fiber bridging. Compared with manual observation, digital sampling provides a higher-resolution description of local morphology and reveals damage gradients that are difficult to quantify visually.

In densely cracked or intersecting regions, pixel resolution may increase the dispersion of individual width measurements. The method is therefore better suited to evaluating crack spacing, width distributions, and damage-evolution trends than to resolving every local width at subpixel scale.

#### 3.2.4. Correlation of Visual and Measured Strain

**Figure 14:**
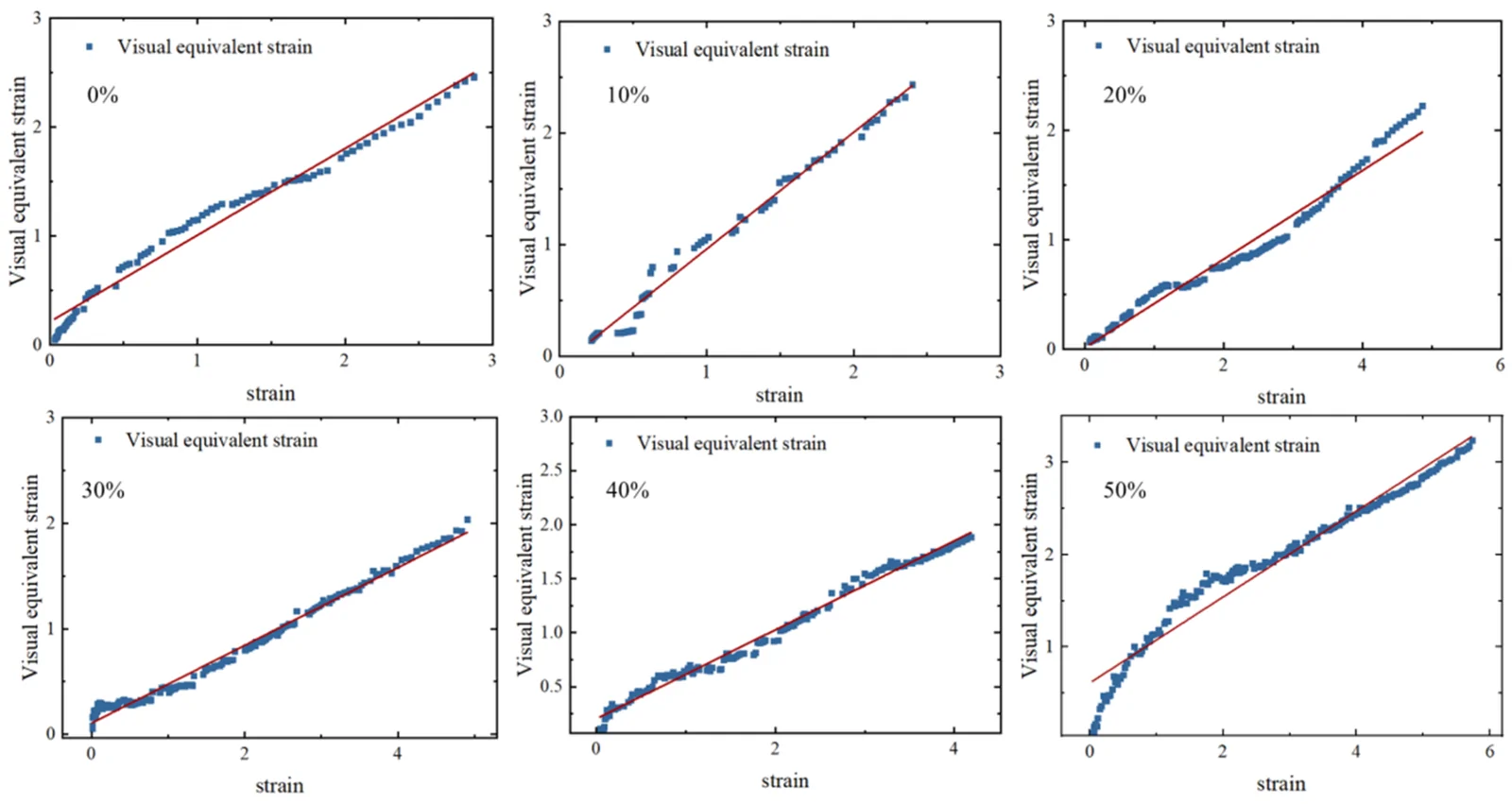
Linear regressions between tensile strain and visual equivalent strain for ECC with rubber replacement ratios of 0–50%. The blue line indicates the location selected for crack-width measurement.

**Figure 15:**
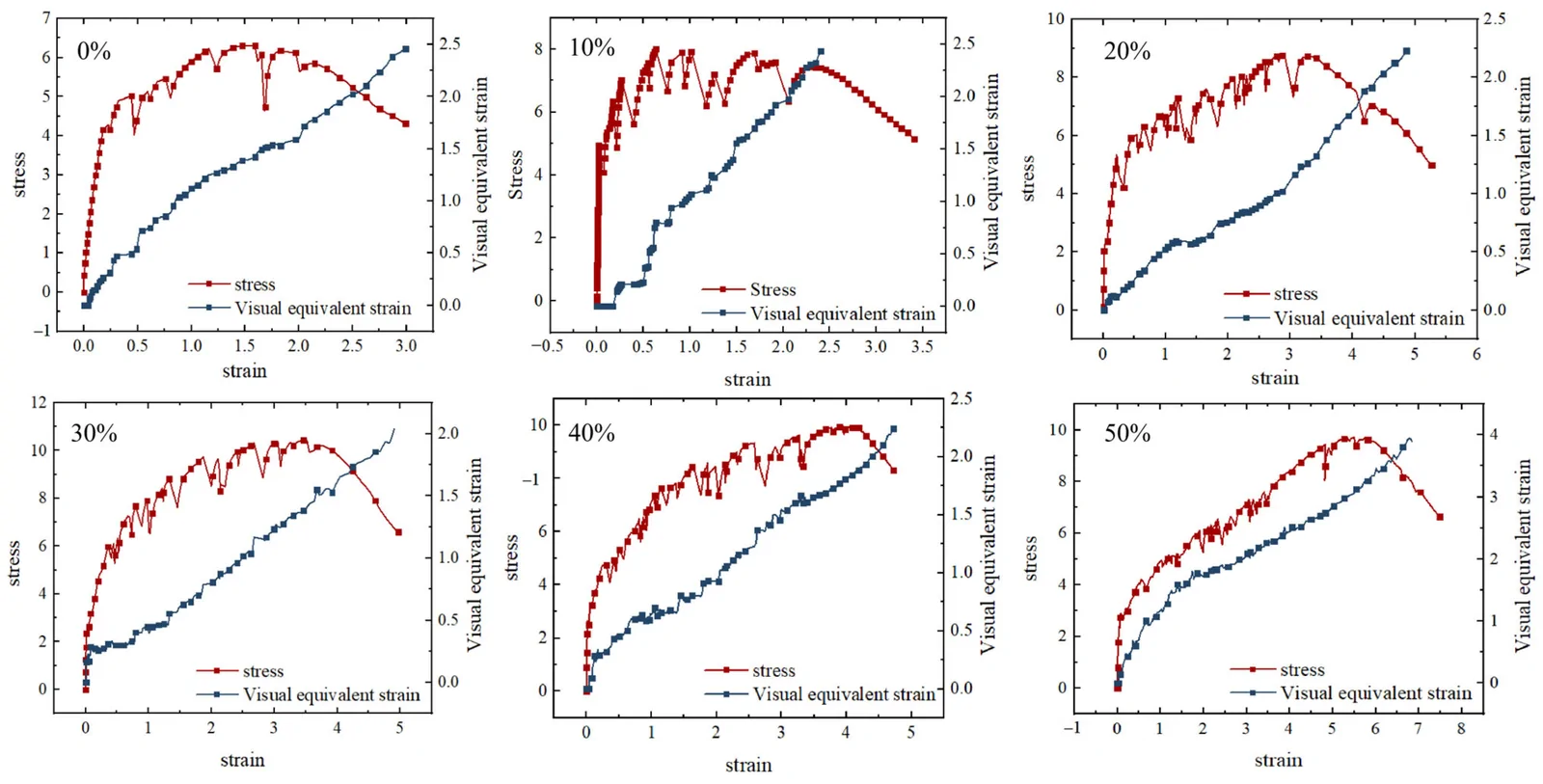
Concurrent evolution of tensile stress and visual equivalent strain for ECC with rubber replacement ratios of 0–50%.

To evaluate whether segmented surface crack geometry can track macroscopic tensile deformation, the crack-area-based visual equivalent strain (*ε_v_*, Equation (22)) was compared against extensometer (LVDT) strain during the multiple-cracking stage (excluding the initial uncracked elastic stage, where matrix elastic strain is below the optical resolution limit). As shown in Table 11 and Figure 14, *ε_v_* exhibited a strong linear correlation with measured tensile strain across all six rubber replacement ratios (0–50%), with Pearson’s *r* ranging from 0.974 to 0.994 and *R*^2^ ranging from 0.948 to 0.989. Furthermore, plotting *ε_v_* alongside the tensile stress–strain curves (Figure 15) demonstrates that the optical indicator accurately captures the micromechanical transition of rubberized ECC: at low rubber contents (0–20%), *ε_v_* grows in discrete steps synchronized with sudden stress drops during individual crack formation; at higher rubber contents (30–50%), as matrix toughness decreases and microcracking becomes finer and more frequent [64,66], both the stress–strain curve and the *ε_v_* trajectory transition into smooth, continuous strain-hardening curves.

Despite the consistently high linear correlation, correlation alone does not establish quantitative measurement agreement. As shown in Table 11, while the linear regression slope is close to unity for the 10% rubber mixture (1.046) and moderate for the 0% mixture (0.796), it decreases substantially to 0.368–0.465 for the 20–50% rubber replacement groups. To rigorously quantify absolute discrepancies, root-mean-square error (RMSE), mean absolute error (MAE), and Bland–Altman agreement metrics (mean bias and 95% limits of agreement, LoA) between uncalibrated *ε_v_* and measured strain were computed across paired loading observations (Table 11). While the 10% rubber group showed minimal uncalibrated error (RMSE = 0.03%, MAE = 0.03% strain), the uncalibrated RMSE and MAE increased to 1.18–2.34% and 0.99–2.04% strain, respectively, for the 20–50% rubber groups, accompanied by negative Bland–Altman biases (−0.99% to −2.03% strain).

Figure 15 further compares the visual measurements with the mechanical response. The visual equivalent-strain curves captured the transition from discrete multiple cracking to dense crack saturation.

At low rubber replacement ratios of 0–20%, visual equivalent strain increased in steps, and individual jumps generally coincided with stress drops. The visual method therefore captured sudden propagation of discrete cracks.

As the rubber content increased, more cracks formed and individual cracks became narrower, gradually producing a saturated crack network—a transition consistent with the reduced matrix fracture toughness and the crack-activation effect of low-modulus rubber inclusions reported for rubberized ECC [64,65,66] (Section 3.2.2). The visual equivalent-strain response consequently changed from stepwise growth to a smoother, approximately linear increase. This transition was consistent with the weaker stress fluctuations caused by individual cracking events at higher rubber contents.

This systematic underestimation of absolute strain magnitude at higher rubber replacement ratios stems from three physical and optical limitations inherent to defining *ε_v_* solely from 2D segmented crack area:

(1) Uncracked matrix compliance: Incorporating 20–50% powdered rubber substantially lowers the elastic modulus of the cementitious matrix [64,66], increasing the elastic and local viscoelastic elongation carried by intact matrix segments between cracks—a deformation captured by the LVDTs but absent from binary crack masks.
(2) Unresolved subpixel and nascent microcracks: As shown in Section 3.2.2, high rubber contents reduce the mean crack width to ~107 μm and generate numerous hair-like microcracks. Microcracks with openings narrower than the physical pixel size (~17 μm) or areas below the 200-pixel denoising threshold remain undetected, causing the segmented area *A_C_* to underestimate the true cumulative crack opening.
(3) Idealized 2D geometric assumptions: Equation (22) assumes that cracks are strictly perpendicular to the loading axis and open uniformly across the full gauge width B and specimen thickness. In practice, higher crack tortuosity, non-penetrating branch cracks, and three-dimensional through-thickness bridging variations cause the single-surface 2D area fraction to deviate from volumetric axial extension.

Consequently, *ε_v_* should be interpreted as a high-sensitivity optical damage-evolution proxy rather than an uncalibrated direct strain sensor; when quantitative strain estimation is required, mixture-specific linear calibration (scaling by the inverse regression slope) must be applied.

### 3.3. Integrated Application of the Intelligent Crack-Analysis System

To demonstrate practical integration of the model and quantitative metrics, an ECC crack inspection and analysis platform was developed separately in Python 3.8 using PyQt5. Model training and ablation experiments used Python 3.11 (Section 2.6), whereas the standalone PyQt5 desktop platform was packaged in Python 3.8 to ensure compatibility with legacy GUI dependencies. The platform combines image segmentation, feature quantification, and result export, automating the conversion of raw tensile images into crack-parameter reports.

#### 3.3.1. Crack-Detection and Quantification Results

The crack-analysis module contains semantic-segmentation and crack-quantification submodules. It processes images in the sequence of input, segmentation, post-processing, scale conversion, and parameter output, converting each raw image into structured crack metrics.

**Figure 16:**
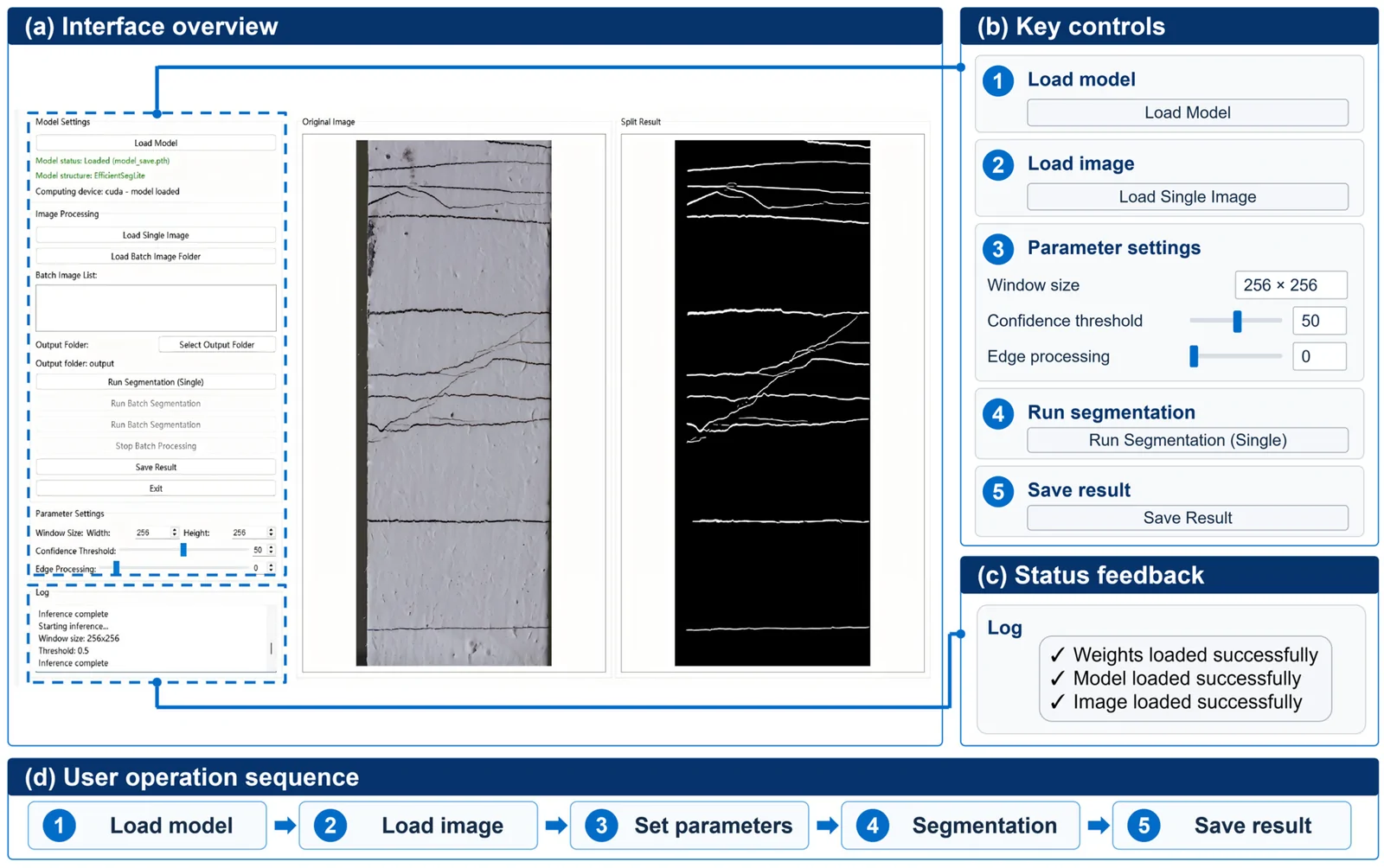
Graphical user interface and operational workflow of the semantic-segmentation submodule: (**a**) interface overview showing the control panel, original specimen image, and binary segmentation result; (**b**) enlarged view of the key interactive controls for model loading, image import, sliding-window and threshold configuration, inference execution, and result export; (**c**) status feedback log displaying runtime execution messages; and (**d**) five-step user operation sequence.

The semantic-segmentation submodule incorporates EfficientSegLite, as shown in Figure 16. High-resolution images are divided using a sliding window, predicted patch by patch, and reassembled at their original coordinates. Predictions in overlapping regions are combined by arithmetic averaging to reduce seam artifacts. The interface supports weight loading, image preview, and segmentation-result display and provides binary masks for subsequent quantification.

**Figure 17:**
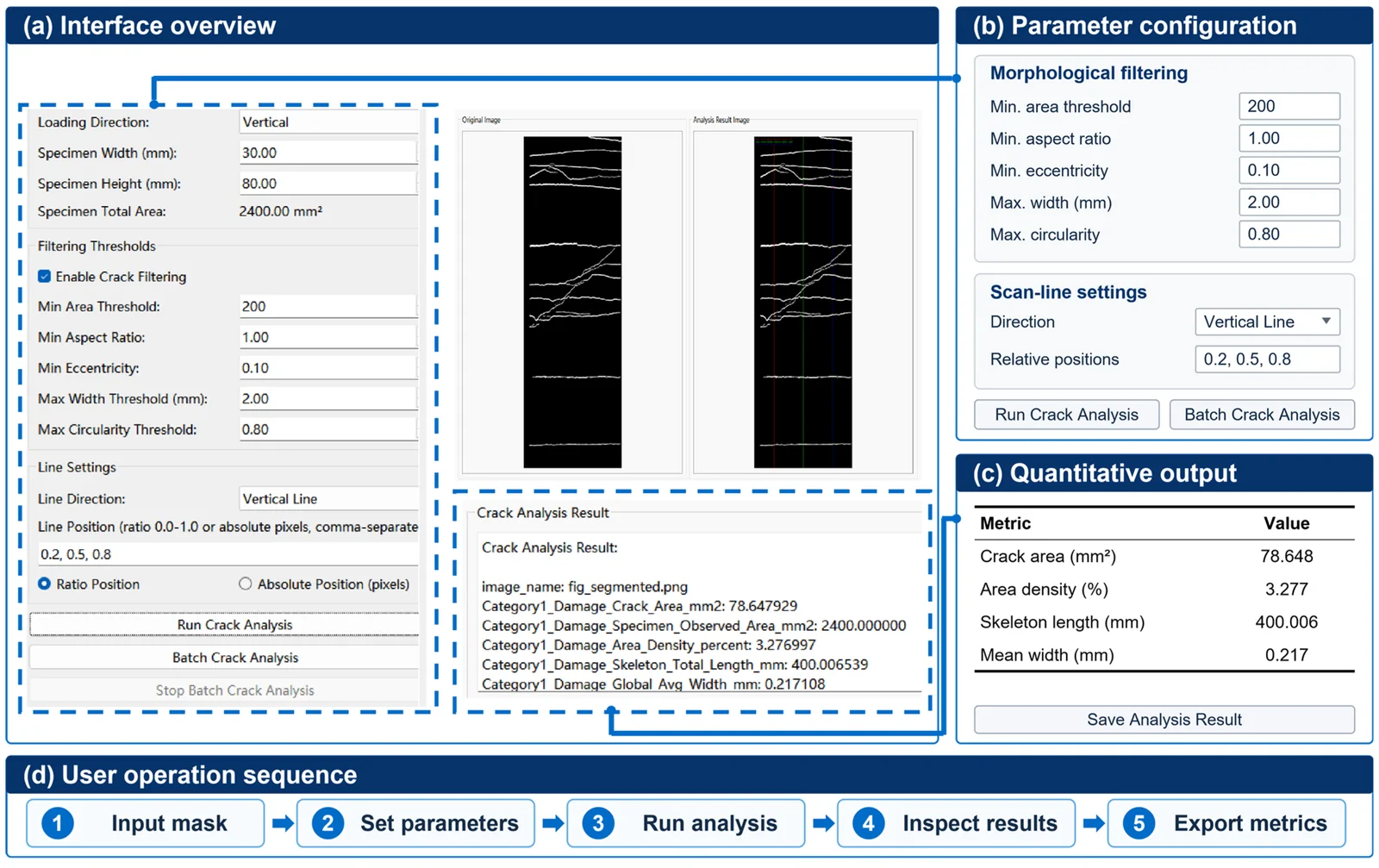
Graphical user interface and operational workflow of the crack-quantification submodule: (**a**) interface overview displaying the parameter panel, binary mask input, scan-line visualization, and raw output log; (**b**) enlarged view of the configurable morphological filtering thresholds and scan-line settings; (**c**) structured summary of extracted physical crack descriptors (crack area, area density, skeleton length, and mean width); and (**d**) five-step user operation sequence from mask input to metric export.

The crack-quantification submodule receives the binary mask, as shown in Figure 17. Connected components are first labeled, and an area threshold removes false regions caused by pores, fiber texture, and imaging noise. The crack skeleton, boundary, and branch nodes are then extracted. After scale mapping between the observed physical area and image resolution, the system reports crack count, total length, mean and maximum width, area density, and morphological-complexity metrics.

#### 3.3.2. Results from the Large Language Model Agent

The agent uses a Streamlit interface with a console, result-analysis region, and natural-language input window. The console configures the inference mode, data path, and batch-processing tasks. The input window accepts uploaded images or text questions, and the result region displays quantitative crack metrics and analytical text. Users can invoke crack-detection and quantification functions through natural-language instructions.

**Figure 18:**
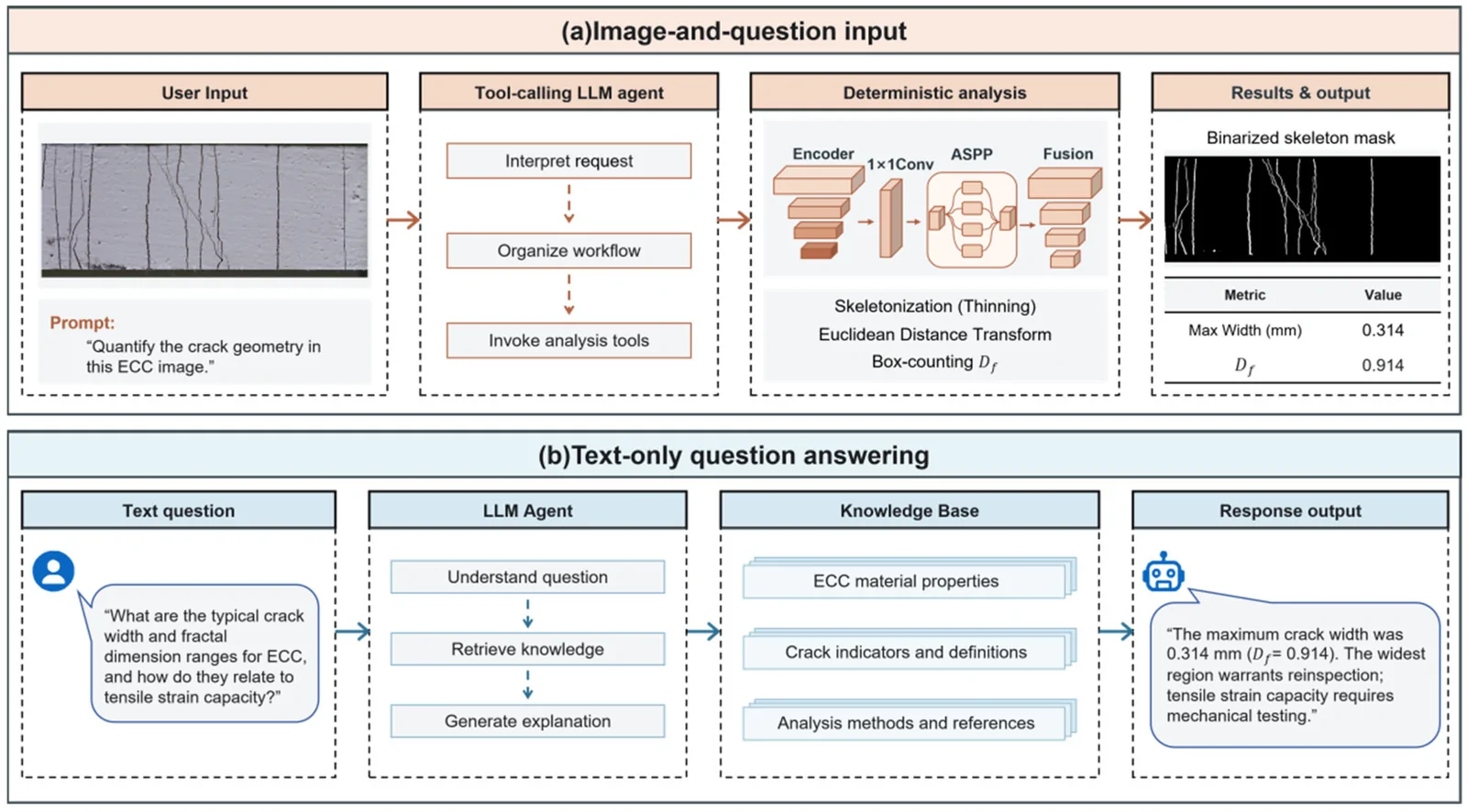
Workflow of the intelligent agent system: (**a**) image-and-question input for crack analysis; (**b**) text-only knowledge consultation. EfficientSegLite performs crack segmentation, while deterministic algorithms compute crack metrics. The large language model handles instruction interpretation, workflow orchestration and text generation.

The agent supports joint image-and-question input and text-only question answering, as illustrated in Figure 18. In the joint-input mode, it invokes EfficientSegLite and the crack-quantification program to perform detection, parameter extraction, result aggregation, and report generation. The text-only mode explains ECC properties, crack metrics, and analytical methods. Deterministic algorithms calculate every crack metric, while the LLM is limited to instruction interpretation, workflow organization, and text generation.

## 4. Conclusions

This study developed and evaluated an EfficientSegLite-based framework for lightweight segmentation and physical quantification of ECC cracks. The framework combines segmentation accuracy, computational efficiency, and automated crack characterization. The main conclusions are as follows:

(1) EfficientSegLite combines a SegFormer-B0 encoder, a modified U-Net decoder, Light-ASPP, and DW-Conv for efficient segmentation of fine cracks. It achieved an mIoU of 93.40% and an F1 score of 93.24%, with 734.9 M FLOPs and 3.96 M parameters.
(2) The Dice-OHEM-Edge composite loss uses two-stage weighting. It promotes regional convergence early in training and later emphasizes hard pixels and crack boundaries, improving boundary integrity and segmentation stability in complex backgrounds.
(3) The segmented masks support automated calculation of crack length, width, area density, tortuosity, and fractal dimension. Total crack area was further converted into visual equivalent strain. Across rubber replacement ratios of 0–50%, Pearson’s *r* exceeded 0.97 and *R*^2^ exceeded 0.94. Visual equivalent strain reliably tracked damage during multiple cracking, although estimation of its absolute magnitude requires further calibration.
(4) A model-agnostic, tool-calling agent connects natural-language parsing, deterministic crack analysis, and structured report generation. Local algorithms calculate all crack metrics, while the LLM only organizes the workflow and expresses the results, improving traceability.

Despite its effectiveness, several limitations of the present study should be acknowledged to guide future work. First, the internal dataset partition was conducted at the patch level across 15 specimens; although cross-domain evaluations on DeepCrack and Hao-ECC confirmed external generalization, future studies should expand the specimen cohort to implement strict specimen-level k-fold cross-validation. Second, optical crack-width accuracy depends on physical pixel resolution (~17 μm/px), segmentation boundary fidelity, connected-component filtering (200-pixel threshold), and planar ROI scale calibration without intrinsic lens-distortion rectification; moreover, the in situ optical microscopy validation was limited to eight representative cracks. Subpixel microcracks (<20–30 μm) and dense branching intersections therefore remain challenging to resolve individually. Third, because the visual equivalent strain omits uncracked matrix deformation and subpixel cracks, it underestimates absolute tensile strain at high rubber contents (20–50%) and requires mixture-specific calibration for quantitative strain prediction. Future work will integrate intrinsic camera calibration, large-scale microscopic validation, and multi-modal sensing (e.g., digital image correlation, acoustic emission, and X-CT) for three-dimensional coupled micromechanical analysis.

## Figures and Tables

**Table 1:** Comparison of representative studies on crack segmentation and geometric characterization.

Study	Dataset/Application	Method	Key Findings/Contribution
Liu et al. [42]	DeepCrack: 537 annotated images of cracks in multiple scenes.	Hierarchical convolutional network with deep supervision and multiscale feature aggregation.	Combines information across feature levels for pixel-wise crack segmentation.
Wang and Su [36]	Public dataset of 458 high-resolution building images from Middle East Technical University.	Hierarchical transformer encoder; top-down decoder with lateral connections; online hard example mining.	Integrates multiscale context and hard-example selection for concrete-crack segmentation.
Carrasco et al. [39]	Plain and fiber-reinforced earthen construction materials; natural and artificial lighting.	Anisotropic smoothing, adaptive segmentation, stabilized clustering, and orientation-aware width profiles.	Measures local crack width while accounting for changes in crack orientation.
Li et al. [40]	Asphalt-pavement crack images with a calibration object for physical scale.	Image enhancement, Sauvola binarization, connected-component filtering, and Zhang–Suen thinning.	Extracts crack area, width, length, and segments using calibrated image processing.
Hao et al. [43]	ECC multiple-crack images; unrelated images for generalization assessment.	Fully convolutional network with a dual pre-modification strategy.	Targets dense, fine ECC cracks and supports quantitative crack-pattern analysis.
Present study	Images acquired during uniaxial tensile testing of rubberized ECC.	EfficientSegLite segmentation, calibrated geometric analysis, and LLM-assisted tool coordination.	Integrates crack descriptors with evaluation of a crack-area-based indicator against measured tensile strain.

**Table 2:** Physical and mechanical properties of the PE fibers.

Fiber Type	Diameter (μm)	Length (mm)	Tensile Strength (MPa)	Elastic Modulus (GPa)	Elongation (%)	Density (g/cm^3^)
PE	20	12	3000	116	3.5–4	0.97

**Table 3:** ECC mixture proportions (kg/m^3^).

Mixture ID	C	GGBFS	SF	QP	PR	W	SP	PE Fiber
0-12	887	648	133	532	0	258.5	35	19.4
10-12				479	22			
20-12				426	45			
30-12				373	67			
40-12				320	90			
50-12				267	112			

Note: C = P.II 52.5R cement; GGBFS = ground granulated blast-furnace slag; SF = silica fume; QP = quartz powder; PR = powdered rubber; W = water; and SP = polycarboxylate-based high-range water-reducing admixture (solid content 40%; dosage 1.6% by mass of binder). The designation 10-12 indicates a 10% volumetric replacement of quartz powder by powdered rubber and a PE fiber length of 12 mm.

**Table 4:** Loss-weight schedule for the two training stages.

Stage	Epoch Range	Dice-Loss Weight	Focal-Loss Weight	Edge-Loss Weight	OHEM Ratio
1	1–36 (adaptive; max 50)	0.7	0.3	0.0	1.0
2	37–100	0.6	0.3	0.1	0.7

**Table 5:** Loss-function parameter settings.

Loss Component	Parameter	Symbol	Value
Dice Loss	Smoothing constant	*ε*	1 × 10^−6^
Focal Loss	Positive-class weight	α	0.5
	Focusing parameter	γ	2.0
OHEM	Hard-pixel ratio	*r*	0.7
Edge Loss	Edge operator	-	Sobel
	Distance metric	-	L1 (MAE)

**Table 6:** Training hyperparameters and optimization settings.

Hyperparameter	Value	Hyperparameter	Value
Training epochs	100	Learning-rate scheduler	ReduceLROnPlateau
Batch size	8	Reduction factor	0.8
Decoder and head learning rate	1 × 10^−3^	Scheduler patience	20 epochs
Encoder learning rate	1 × 10^−4^	Weight decay	1 × 10^−4^

**Table 7:** Visual parameters for ECC crack characterization.

Category	Parameter	Symbol	Description	Unit
Area	Crack area	*A_C_*	Total projected area of all crack regions within the image field of view; characterizes the overall extent of cracking.	mm^2^
Length	Total crack length	*L*	Total length of the crack network.	mm
Width	Mean crack width	$\bar{w}$	Mean opening width evaluated along the crack centerline; represents the overall crack-opening level.	μm
Complexity	Tortuosity	*τ*	Describes crack-path curvature and propagation complexity.	—
Complexity	Fractal dimension	*D_f_*	Describes the spatial complexity and self-similarity of the crack network, including its density and roughness.	—
Macroscopic inference	Visual equivalent strain	*ε_v_*	Equivalent axial deformation inferred from crack geometry; quantifies the contribution of cracking to strain.	—

**Table 8:** Comparison of the training strategies.

Training Strategy	MPA	mIoU	Recall	Precision	F1 Score
Single-stage (*n* = 3)	97.39 ± 0.56	92.81 ± 0.63	95.34 ± 1.19	90.19 ± 2.69	92.54 ± 0.82
Two-stage (*n* = 3)	96.47 ± 0.27	93.39 ± 0.04	93.35 ± 0.59	93.47 ± 0.55	93.24 ± 0.04

**Table 9:** Performance comparison among segmentation networks.

Network	MPA	mIoU	Recall	Precision	F1 Score	FLOPs	Parameters
U-Net	96.33	91.98	93.26	90.55	91.21	47.09 G	28.60 M
SegFormer-B1	89.80	85.26	80.27	88.02	83.39	2.13 G	13.16 M
SegFormer-B2	90.32	86.37	81.20	89.90	85.31	4.14 G	24.20 M
SegFormer-B2 + U-Net	96.20	92.93	93.39	92.42	92.90	10.12 G	41.37 M
DeepLabV3+	95.49	91.31	94.01	90.10	90.64	45.77 G	40.35 M
SegFormer-B0	90.33	85.93	81.29	88.60	84.78	563.87 M	3.32 M
DDRNet-23-slim	96.09	87.89	93.44	81.81	87.02	1.145 G	5.70 M
BiSeNet V2	93.76	89.79	88.04	90.88	89.11	8.37 G	4.39 M
EfficientSegLite	96.78	93.40	94.01	92.85	93.24	734.9 M	3.96 M

**Table 10:** Ablation results for the components of EfficientSegLite.

Model Variant	Parameters	FLOPs	mIoU	F1 Score
Baseline (SegFormer-B0)	3.32 M	2.188 G	59.71	36.61
+U-Net	4.16 M	4.138 G	92.76	92.33
+U-Net + ASPP	6.62 M	4.659 G	92.83	92.41
+U-Net + ASPP + OptimizedDWConv	3.96 M	734.87 M	93.40	93.24

**Table 11:** Correlation and agreement analysis between visual equivalent strain and extensometer strain at different rubber replacement ratios.

	0%	10%	20%	30%	40%	50%
slope	0.796	1.046	0.405	0.368	0.41	0.465
Pearson’s *r*	0.989	0.993	0.983	0.993	0.994	0.974
*R* ^2^	0.977	0.986	0.966	0.987	0.989	0.948
RMSE (% strain)	0.31	0.03	1.18	1.76	1.91	2.34
MAE (% strain)	0.25	0.03	0.99	1.50	1.67	2.04
Bland–Altman mean bias (% strain)	−0.25	0.01	−0.99	−1.50	−1.67	−2.03
Bland–Altman 95% LoA (% strain)	[−0.63, 0.13]	[−0.05, 0.08]	[−2.32, 0.34]	[−3.40, 0.39]	[−3.54, 0.21]	[−4.34, 0.28]

## Data Availability

The PyTorch implementation of EfficientSegLite, the two-stage Dice–OHEM–Edge loss module, the deterministic morphological quantification scripts, and the model configuration files will be made publicly available on GitHub at [https://github.com/sherlovk111-cyber/EfficientSegLite-ECC.git, accessed on 20 October 2026] upon publication. The full-resolution raw tensile image sequences are available from the corresponding author upon reasonable request.
